# Multi-omics analysis reveals ultraviolet response insights for immunotherapy and prognosis

**DOI:** 10.3389/fimmu.2025.1598070

**Published:** 2025-09-26

**Authors:** DanHua Zhang, Mei Dai, JiaFei Ying, YiFan Huang, ZiXuan Liu, ChenLu Wu

**Affiliations:** ^1^ Department of General Surgery, The Second Xiangya Hospital, Central South University, Changsha, Hunan, China; ^2^ Clinical Research Center for Breast Disease of Hunan Province, Changsha, Hunan, China; ^3^ Department of General Surgery, Shaoxing Maternity and Child Health Care Hospital, Shaoxing, Zhejiang, China; ^4^ Department of Cardiology, The Second Xiangya Hospital, Central South University, Changsha, Hunan, China

**Keywords:** ultraviolet light, immune checkpoint inhibitor, single-cell sequencing, bulk-RNA seq, pan-cancer

## Abstract

**Background:**

Immune checkpoint inhibitors (ICIs) have revolutionized cancer immunotherapy, but many patients develop resistance. While the immunosuppressive effects of ultraviolet (UV) light are well-documented, its link to ICI resistance remains unclear.

**Methods:**

We analyzed publicly available single-cell RNA sequencing (scRNA-seq) datasets from ICI-treated patients to explore the relationship between UV response (UVR) and treatment outcomes. A novel UVR gene signature (UVR.Sig) was established using 34 scRNA-seq datasets and validated in The Cancer Genome Atlas (TCGA) pan-cancer cohorts and 10 ICI cohorts. Key genes (Hub-UVR.Sig) were identified via six machine learning algorithms, and breast cancer (BRCA) subtypes were classified through consensus clustering. Biological effects of Hub-UVR.Sig genes were confirmed *in vitro*.

**Results:**

UVR.Sig was associated with ICI resistance and correlated with inhibitory immune cell infiltration and pro-tumor pathways in pan-cancer data. The UVR.Sig-based model achieved good predictive performance for ICI outcomes (AUC = 0.727). In BRCA, Hub-UVR.Sig stratified patients into two subtypes, with high Hub-UVR.Sig expression linked to stronger immune evasion and lower immunogenicity. ENO2 and ATP6V1F were highly expressed in BRCA tissues, and ENO2 was correlated with worse prognosis in BRCA patients. Knockdown of ENO2 reduced cell proliferation and invasion.

**Conclusion:**

We reveal for the first time that UVR is strongly associated with ICI resistance. The UVR.Sig feature offers the potential to identify patients who respond to immunotherapy and to tailor BRCA treatment strategies.

## 1 Introduction

Significant progress has been made in understanding the critical role of immune checkpoint inhibitors (ICIs) in regulating the activity of tumor-infiltrating T cells, leading to a revolutionary shift in cancer immunotherapy (1, 2). Immunotherapeutic modalities including ICIs, vaccination and passive cell transfer have been extensively studied in the clinical setting of breast cancer (BRCA), particularly in patients with triple negative breast cancer (TNBC) (3). However, only a small subset of patients benefited from immunotherapy, with the majority experiencing either primary or acquired resistance (4–7). Therefore, identifying appropriate biomarkers for ICI therapy sensitivity is crucial for optimizing treatment options and improving patient outcomes.

Ultraviolet (UV) light at wavelengths ranging from 10 to 380 nm is a form of electromagnetic radiation. The process by which cells or organisms undergo changes in their activity or state (such as movement, secretion, enzyme production, and gene expression) in response to UV exposure is the UV response (UVR). Although the immunosuppressive effects of UVR have been well established, direct evidence linking UVR to immunotherapy response remains unavailable. Early studies have demonstrated that chronic UV exposure modulates immune and antigenic responses, influencing the carcinogenic process in the skin (8). This discovery spurred further investigations into the mechanisms underlying UVR-induced immunosuppression. Subsequent research revealed that UV-induced DNA damage, reactive oxygen species generation, Treg induction, and the release of immunosuppressive cytokines, such as IL-10, are closely associated with UVR-mediated immunosuppression (9, 10). Recent studies have suggested that UVR promotes immunosuppression by regulating the expression of immune checkpoints. UV exposure activates the IRF3 and NF-κB pathways via HMGB1, leading to the upregulation of PD-L1 expression and reduction in tumor cell sensitivity to CD8+ T cell-mediated cytotoxicity (11). Although Carlos et al. identified a UVR-related gene signature that underscores the association between UVR and inhibitory immune cells, including immature dendritic cells, plasmacytoid dendritic cells, and M2 macrophages, within the immunosuppressive microenvironment of uveal melanoma (12), there is still a substantial gap in the literature regarding the role of UVR-related genes in tumors and their impact on immunotherapy outcomes.

The single-cell RNA sequencing (scRNA-Seq) technique enables the dissection of complex interactions between tumor cells and immune cells at the single-cell level, facilitating a better understanding of the dynamic mechanisms underlying tumor-immune interactions (13–16). This study combines scRNA-seq technology with comprehensive bioinformatics analysis with the aim of constructing a predictive model of ICI efficacy based on the expression profiles of UVR-associated genes, laying the groundwork for improved stratification and personalized treatment of tumor patients. In addition, this study bridges a significant gap in our understanding of the role of UVR-associated genes in BRCA.

## 2 Methods and materials

### 2.1 Identification of UVR-related genes

UVR-related genes were collected from the Molecular Signatures Database (MSigDB) (17). A search using the keyword “UV Response” in MSigDB yielded 191 genes included in the HALLMARK_UV_RESPONSE dataset (The complete gene list was provided in **Supplementary Table S1**).

### 2.2 Pan-cancer transcriptomic dataset and processing

The pan-cancer transcriptomic dataset from The Cancer Genome Atlas (TCGA) was obtained from the UCSC Xena platform (https://xenabrowser.net) (18) to investigate the potential association between UVR-related genes and immune suppression across 30 cancer types. To avoid interference from the dominant effects of immune cells, three cancer types primarily composed of immune cells were excluded: acute myeloid leukemia, diffuse large B-cell lymphoma, and thymoma. Additionally, tumor mutational burden (TMB) and microsatellite instability (MSI) data were obtained from the cBioPortal database (19) for subsequent analysis. Relevant clinical and pathological information for the 30 cancer types were downloaded using the R package TCGAbiolink (20). Patients included in the analysis met the following criteria: availability of mRNA expression and clinical data, completion of standard diagnosis and treatment, and a survival time longer than 30 d.

### 2.3 Acquisition and processing of ICI-related datasets

To investigate the relationship between UVR-related genes and immunotherapy, the R package GEOquery (21) was used to download two scRNA-seq datasets with well-defined efficacy for tumor immunotherapy from the GEO database. The Gene Set Variation Analysis (GSVA) R package (22) was employed to assess the enrichment scores of UVR-related genes in these datasets and explore their association with ICI efficacy. These two datasets were the skin cutaneous melanoma (SKCM, GSE115978) (23) and the basal cell carcinoma (BCC, GSE123813) datasets (24). After quality control (QC), the SKCM dataset GSE115978 included 32 patients, comprising 15 non-responders (NRs) who did not benefit from immunotherapy, 16 treatment-naïve patients (TN) who did not undergo immunotherapy, and 1 responder who responded to treatment.

Ten bulk RNA-seq datasets related to ICI treatment were systematically collected. These datasets included five SKCM datasets [Hugo 2016 (25), Liu 2019 (26), Gide 2019 (27),Riaz 2017 (28) and Van 2015 (29)], two urothelial carcinoma (UC) datasets [Mariathasan 2018 (30) and Synder 2017 (31)], one GBM dataset [Zhao 2019 (32)], one renal cell carcinoma (RCC) dataset [Braun 2020 (33)], and one gastric cancer (GC) dataset [Kim 2018 (34)]. The Hugo 2016 SKCM dataset consisted of 27 preprocessed tumor samples from 26 patients, while the GBM dataset included 34 preprocessed tumor samples from 17 patients. For both datasets, one tumor sample per patient was randomly selected for analysis.

### 2.4 Collection of published signatures for comparison

Six pan-cancer signatures [INFG.Sig (35), T.Cell. Infamed.Sig (35), PDL1.Sig (36), LRRC15.CAF.Sig (37), NLRP3.Sig (38) and cytotoxic.Sig (39)] were gathered along with four SKCM-specific signatures [CRMA.Sig (40), IPRES.Sig (25), IMS.Sig (41) and TRS.Sig (42)]. The codes and algorithms for these 10 signatures were obtained from their original studies, such as single-sample gene set enrichment analysis (ssGSEA) for NLRP3.Sig and cancer classification for ImmuneCell.Sig.

### 2.5 Collection and processing of scRNA-seq data

We collected 34 scRNA-seq datasets containing stromal or immune cells from the TISCH database (43), comprising 345 patients and 663,760 cells across 17 cancer types. These cancer types included BCC, BRCA, multiple myeloma (MM), neuroendocrine tumor (NET), non-small cell lung cancer (NSCLC), ovarian serous cystadenocarcinoma (OV), pancreatic adenocarcinoma (PAAD), SKCM, stomach adenocarcinoma (STAD), uveal melanoma (UVM), cholangiocarcinoma (CHOL), colorectal cancer (CRC), GBM, head and neck cancer (HNSC), liver hepatocellular cancer (LIHC), medulloblastoma (MB), and Merkel cell carcinoma (MCC).

The integration and analysis of scRNA-seq data were performed using the Seurat v4.0.6 R package (44), with doublet QC conducted using the R package Scrublet v0.2 (45). During QC, cells with fewer than 300 detected genes and those with mitochondrial gene reads exceeding 20% of the total reads were excluded. Data normalization and standardization were performed using principal component analysis (PCA) (46), and batch effects across samples were corrected using the Harmony R package (47).

### 2.6 Generation of UVR.Sig

A UVR gene signature (UVR.Sig) was generated by calculating the enrichment scores of UVR-related genes across various scRNA-seq datasets using the GSVA R package. Spearman’s correlation analysis was conducted between the expression levels and enrichment scores of UVR-related genes, marking the positively correlated genes (Spearman r > 0.3 and p < 0.05) as Gx. The “FindMarkers” function was used to identify differentially expressed genes (DEGs) in malignant tumor cells from each scRNA-seq dataset, where genes with |logFC| ≥ 0.30 and FDR (q value) < 1e-05 were considered upregulated DEGs in malignant tumor cells and labeled as Gy. To obtain the upregulated tumor-specific DEGs that were positively correlated with UVR, the intersection of Gx and Gy was considered for each dataset to generate the gene set, Gn. Finally, the Gn genes from all datasets were combined and deduplicated to form the UVR.Sig.

### 2.7 Gene Ontology and Kyoto Encyclopedia of Genes and Genomes pathway enrichment analysis

GO (48) analysis is commonly used for functional enrichment studies, encompassing biological processes (BP), cellular component (CC), and molecular function (MF). KEGG (49) is a widely used database that provides information on genomes, biological pathways, diseases, and drugs. GO and KEGG enrichment analyses were performed on the UVR.Sig using the ClusterProfiler R package (50). The selection criteria for significant enrichment were set to adj.p < 0.05 and FDR (q value) < 0.25, with p-values adjusted using the Benjamini–Hochberg method.

### 2.8 Immune-related analysis of UVR.Sig

To further evaluate the immune relevance of UVR.Sig, the UVR.Sig scores were first calculated across 30 cancer types from TCGA pan-cancer transcriptome dataset using the GSVA R package. A correlation analysis was performed between the UVR.Sig scores and 75 published immune-related genes (51), and the results were visualized using correlation circle plots. Next, based on the abundance of immune cells, the tumor immune microenvironment was determined across different cancer types. Immune infiltration analysis was conducted using Microenvironment Cell Populations Counter (MCPcounter) (52), which calculated absolute abundance scores for eight immune cells and two stromal cell types from the gene expression matrix. These cells include T cells, CD8+ T cells, cytotoxic lymphocytes, natural killer (NK) cells, B lymphocytes, monocytes, myeloid dendritic cells, neutrophils, endothelial cells, and fibroblasts. The results were visualized by generating a correlation heatmap using the “MCPcounter” function from the IOBR R package (53). Finally, all pathways were obtained from the HALLMARK gene set using the MSigDB database, the correlation between UVR.Sig and each pathway was calculated, and the results were visualized using a bubble plot.

### 2.9 Clinical efficacy evaluation

The primary clinical outcomes included objective response rate (ORR) and overall survival (OS). For all datasets, except Hugo 2016, the ORR was assessed using Response Evaluation Criteria in Solid Tumors (RECIST) version 1.1 (54). The Hugo 2016 dataset utilized immune-related RECIST to evaluate ORR. Based on their response status, the participants were categorized into two groups: responders, which included patients with complete response or partial response, and NRs, which included patients with stable or progressive disease.

### 2.10 Construction of the ICI efficacy prediction model

First, the five ICI RNA-seq datasets with the largest patient samples were combined to form a new, large dataset (n = 772), including RCC (n = 181), UC (n = 348), and SKCM (n = 243) datasets. The five ICI RNA-seq datasets were Braun 2020 RCC, Mariathasan 2018 UC, Liu 2019 SKCM, Gide 2019 SKCM, and Riaz 2017 SKCM. The ComBat method was used to eliminate batch effects among different datasets. The merged dataset was randomly divided into two sets: a training (80%, n = 618) and validation (20%, n = 154) set. The remaining five ICI RNA-seq datasets were used as independent testing sets (n = 149): Zhao 2019 GBM, Snyder 2017 UC, Van Allen 2015 SKCM, Kim 2018 GC, and Hugo 2016 SKCM datasets.

Next, the immune response prediction models were trained using UVR.Sig and a training set using seven common machine learning (ML) algorithms. The seven ML algorithms were: Naive Bayes, Random Forest (RF), Support Vector Machine, AdaBoost Classification Trees, Boosted Logistic Regressions, k-Nearest Neighbors, and the Cancerclass algorithms. For each ML algorithm with parameters, except Cancerclass, hyperparameter tuning was performed using five-fold cross-validation to optimize the model performance. To ensure robustness, the optimization process was repeated 10 times with different random seeds for each resampling. For the parameter-free cancer-class algorithm, the entire training set was directly used to train the model. Subsequently, the area under the curve (AUC) values of the seven models in the validation set were compared to identify the most effective ML algorithm for the final UVR-related ICI efficacy prediction model. An AUC > 0.5 indicated a positive association between molecular expression and event occurrence, and the closer the AUC was to 1, the better the diagnostic performance. Specifically, an AUC between 0.5 and 0.7 suggests low accuracy, between 0.7 and 0.9 indicates moderate accuracy, and above 0.9 indicates high accuracy.

To further compare the predictive performance of UVR.Sig, its AUC values were evaluated against six previously published ICI response signature-related gene sets (PDL1.Sig, LRRC15.CAF.Sig, INFG.Sig, T.cell.infamed.Sig, NLRP3.Sig, and Cytotoxic.Sig) across the training, validation, and five independent test sets. We showed AUC from the training, validation, and three best-performing independent test sets (Synder 2017 UC, Kim 2018 GC, and Hugo 2016 SKCM).

### 2.11 Collection and processing of CRISPR datasets

To identify the potential therapeutic targets for UVR.Sig, data from seven published CRISPR/Cas9 screening studies that assessed the effects of individual gene knockouts on tumor immunity were gathered. These studies included Freeman 2019 (55), Kearney 2018 (56), Manguso 2017 (57), Pan 2018 (58), Patel 2017 (59), Vredevoogd 2019 (60) and Lawson 2020 (61). Based on the model cell lines and the applied treatment conditions, data from the seven CRISPR studies were divided into 17 datasets. CRISPR analysis included cell lines from SKCM, GBM, CRC, and RCC. These data were used to identify genes that were highly likely to regulate lymphocytes and affect ICI response across various datasets.

CRISPR screening was performed by performing whole-genome CRISPR-Cas9 knockouts in various cancer cell lines. Screening was performed in two environments: *in vitro*, where different cancer cell lines were cultured with and without cytotoxic T lymphocytes (CTLs), and *in vivo*, where different cancer cell lines were implanted in immunodeficient or immunocompetent mice. Following these treatments, RNA-seq was performed to evaluate the abundance of the corresponding gene-specific single guide RNA(sgRNA). To measure the impact of gene knockouts on CTLs pressure or anti-tumor immunity, the logFC values between different groups of cell lines were calculated. Normalized z-scores were computed from the logFC values to eliminate batch effects and allow for comparisons across different CRISPR datasets. Lower z-scores indicated a better immune response after gene knockout. Genes were ranked based on the average z-scores from the 17 datasets, with lower z-scores indicating a higher ranking. Genes with the lowest z-scores were considered potential immune resistance genes.

Additionally, to further assess the predictive value of UVR.Sig, it was compared with previously identified signatures used to predict ICI response, including five pan-cancer signatures (INFG. Sig, T.cell.infamed.Sig, PDL1.Sig, LRRC15.CAF.Sig, Cytotoxic.Sig) and four SKCM-specific features (CRMA.Sig, IPRES.Sig, IMS.Sig, and TRS.Sig).

### 2.12 Construction of prognostic risk models using ML algorithms

To explore the relationship between UVR.Sig and the prognosis of patients with cancer, a prognostic risk model of UVR.Sig was constructed using six ML algorithms: Bagged Trees, Bayesian, Learning Vector Quantization (LQV), Wrapper (Boruta), Least Absolute Shrinkage and Selection Operator (LASSO), and RF. Finally, the results of the different algorithms were compared. Genes that appeared in at least four ML algorithms were selected as Hub-UVR.Sig. Kaplan–Meier (KM) analysis was conducted using the R package survival (62), and KM curves for both the training and validation cohorts were generated based on risk scores to compare OS differences between the high- and low-risk groups.

### 2.13 Panoramic analysis of Hub-UVR.Sig

The Hub-UVR.Sig enrichment scores were calculated using the ssGSEA algorithm from the R package GSVA based on TCGA pan-cancer transcriptomic dataset. This analysis aimed to explore the correlation between Hub-UVR.Sig and immune cell infiltration abundance across 30 different cancer types. Subsequently, the relationship between Hub-UVR.Sig and MSI was investigated. Finally, to evaluate the prognostic significance of Hub-UVR.Sig in patients with cancer, the correlation between OS and Hub-UVR.Sig was analyzed across various cancer types and KM curves were constructed. Based on the results of the correlation and KM analyses, we focused on specific cancer types.

### 2.14 Construction and correlation analysis of BRCA subtypes

Consensus Clustering (63) is a resampling-based algorithm used to determine subgroup membership and validate clustering accuracy. Through multiple iterations on the subsamples of the dataset, this method introduces sampling variability, offering stability and metrics for selecting the optimal clustering parameters. Using the consensus clustering method from the R package ConsensusClusterPlus (64),different subtypes of BRCA were identified based on Hub-UVR.Sig. During this process, the number of clusters was set between two and six, with 1000 resampling iterations, extracting 80% of the total samples each time (clusterAlg = KM, distance = Euclidean).

To obtain Tumor Immune Dysfunction and Exclusion (TIDE) scores for different subtypes of BRCA, the TIDE algorithm (65, 66) was applied to the expression matrix of BRCA samples. Differences in TIDE scores were calculated, and differences in MSI and TMB scores were assessed among BRCA subtypes using the Wilcoxon rank-sum test.

### 2.15 Somatic mutation analysis of BRCA subtypes

The “Masked Somatic Mutation” data from TCGA website was selected as the somatic mutation data for BRCA samples and preprocessed using VarScan software. The R package maftools (67) was used to visualize the somatic mutation landscape in the different BRCA subtypes.

### 2.16 Immune infiltration analysis of BRCA subtypes

CIBERSORT (68), utilizing linear support vector regression, was applied to deconvolute the transcriptome expression matrix and estimate the composition and abundance of immune cells in mixed-cell populations. By employing the CIBERSORT algorithm with the LM22 signature gene matrix and filtering the data with immune cell enrichment scores greater than zero, a detailed immune cell infiltration matrix was obtained. The R package ggplot2 was used to create grouped comparison plots to illustrate the differences in immune cell expression among different BRCA subtypes. Additionally, the R package pheatmap was used to generate heatmaps displaying the correlation analysis results between the immune cells and Hub-UVR.Sig across BRCA subtypes. Finally, the correlation between Hub-UVR.Sig and immune cells (p < 0.05) were selected, and correlation scatter plots for the top two positive and top two negative correlations were plotted.

### 2.17 Cell culture, transfection and infection

The human BRCA cell lines MDA-MB-231 and BT549 were purchased from ATCC. Cells were cultured in high-glucose DMEM (Procell, Wuhan, China) supplemented with 10% fetal bovine serum (FBS) (Gibco, USA) and 100 U/mL penicillin/streptomycin (Procell, Wuhan, China) in a sterile incubator at 37 °C with 5% CO2. Tumor cells were seeded into 6-well plates and incubated overnight before transfection with small interfering RNA (siRNA). According to the manufacturer’s protocol, siRNA and negative control (siNC) were transfected into tumor cells using Lipo2000 reagent (Invitrogen, USA). The synthetic sequences for siRNA targeting ENO2 were as follows:

siENO2–1 forward: 5`- GCAACUGUCUGCUGCUCAAGG -3`
siENO2–1 reverse: 5`- UUGAGCAGCAGACAGUUGCAG -3`
siENO2–2 forward: 5`- CGAUGUGUCUGUAUUUCAUGU -3`
siENO2–2 reverse: 5`- AUGAAAUACAGACACAUCGUU -3`
siNC forward: 5`- UUCUCCGAACGUGUCACGUTT -3`
siNC reverse: 5`- ACGUGACACGUUCGGAGAATT -3`

### 2.18 RNA isolation and quantitative real-time RT-PCR

Breast tumors and corresponding paracancerous tissues were collected from six patients from the Department of Breast Surgery at the Second Xiangya Hospital of Central South University. This study was approved by the Ethics Committee of the Second Xiangya Hospital of Central South University (Ethical approval number: K005), and all participants provided written informed consent. Total RNA was extracted from the cells using RNAex Pro reagent (AG21101, Hunan, China) according to the instructions. The concentration and quality of RNA were detected using a spectrophotometer. Reverse transcription was performed using Evo M-MLV kit (AG11705, Hunan, China) according to the instructions. qPCR was performed using 2X Universal SYBR Green Fast qPCR Mix (RK21203, Wuhan, China) and Gentier 96E/96R real-time PCR system (Tianlong, Shanxi, China) (each sample was performed in triplicate). Glyceraldehyde-3-phosphate dehydrogenase (GAPDH) was used as an internal reference gene, and the relative expression level was calculated by the 2^-ΔΔCt method, and three replicates were tested for each sample. The primers were:

GAPDH forward primer:5′- TGACCTGCCGTCTAGAAAAACCT -3′
GAPDH reverse primer:5′- GCTGTTGAAGTCAGAGGAGACCA -3′
ENO2 forward primer:5′- TGCCTGGTCCAAGTTCACAGC -3′
ENO2 reverse primer:5′- CACTGCCCGCTCAATACGTT -3′

### 2.19 Cell function experiment

The proliferative capacity of tumor cells was assessed using CCK-8 assay. Migration, invasive capacity of tumor cells was assessed by wound healing and Matrigel-coated transwell, respectively. In the CCK-8 experiments, cells were seeded into 96-well plates at a density of 1.5 × 10³ cells per well, and cell pfroliferation was evaluated daily for 4 consecutive days using the Cell Counting Kit-8 (NCM Biotech, Suzhou, China). Optical density (OD) was measured at 450 nm. In wound healing assays, cells were seeded in 6-well plates at a density of 4 × 10^^5^ per well and grown to confluence. After removal of unadhered cells by washing with phosphate-buffered saline (PBS), a sterile 20 μL pipette tip was used to create a scratch wound in each well. Images were taken in the same area at 0 h and 48 h, respectively, and the distance of wound closure was measured to show cell migration ability. As for Matrigel-coated transwell, cells (1 × 10^4) were seeded in serum-free medium in 8 μm (Corning Incorporated, 3464, USA) upper chambers coated with Matrigel (Yeason, 40183ES08, Shanghai, China) and lower chambers with medium containing 20% FBS. After 24 hours of incubation, the cells were fixed with formaldehyde. Unattached cells in the upper chamber were carefully wiped away, and cells attached to the membrane were stained and counted with crystal violet.

### 2.20 Statistical analysis

All data processing and analyses in this study were conducted using R software (Version 4.2.0) except for the cell experiments. Continuous variables are presented as mean ± standard deviation. Comparisons between the two groups were performed using the Wilcoxon rank-sum test. Unless otherwise specified, correlations between different molecules were calculated using Spearman correlation analysis. Statistical analyses of cellular experiments were performed using GraphPad Prism 9. Data are expressed as mean ± SD of at least three independent experiments. Statistical significance was analyzed by Student’s t-test (two-tailed). p-values less than 0.05 were considered statistically significant.

## 3 Results

### 3.1 Flow chart

**Figure 1:**
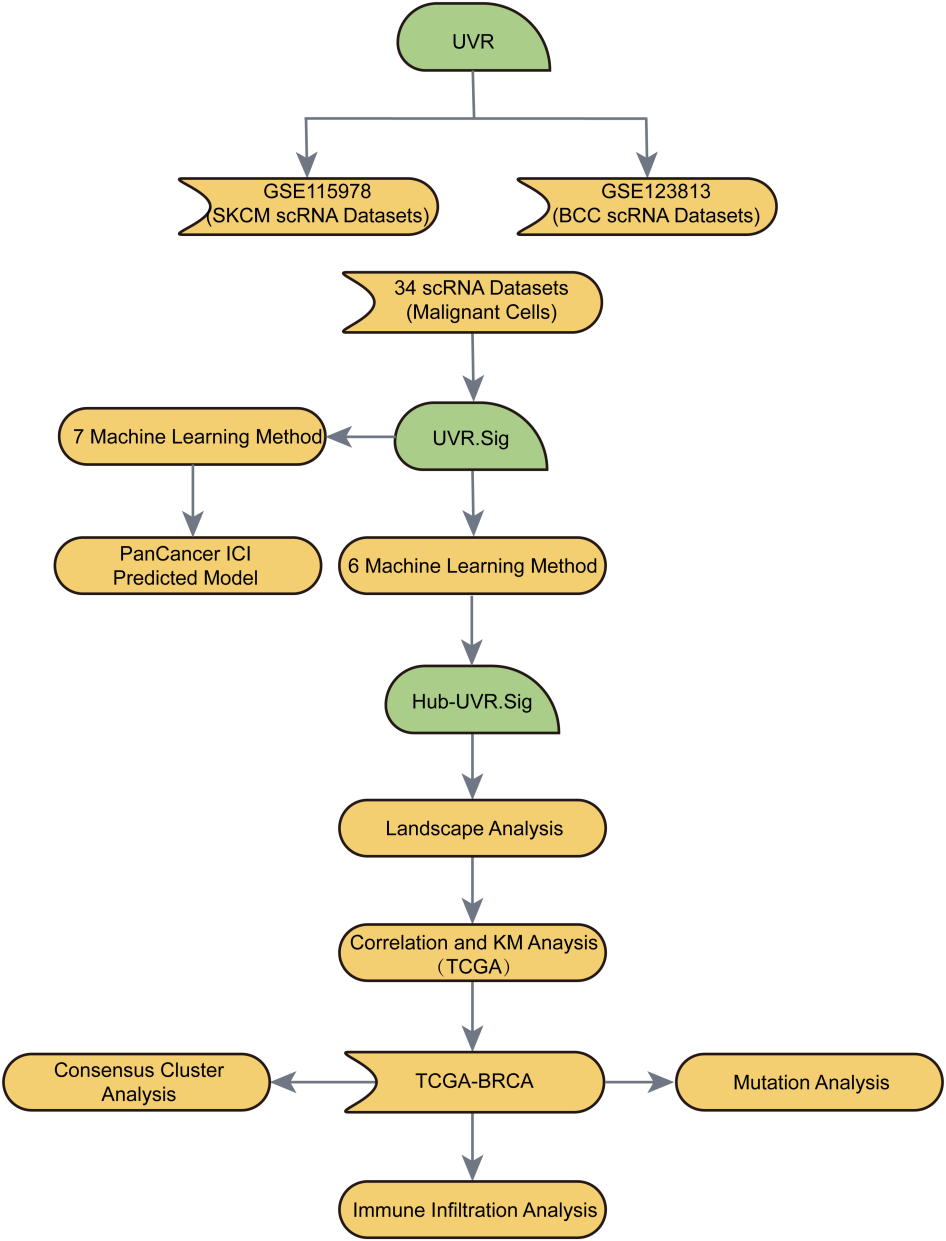
Flow chart for the comprehensive analysis of an ultraviolet response signature (UVR.Sig).

The flow of this study is shown in **Figure 1**.

### 3.2 Negative correlation between UVR-related genes and ICI efficacy

**Figure 2:**
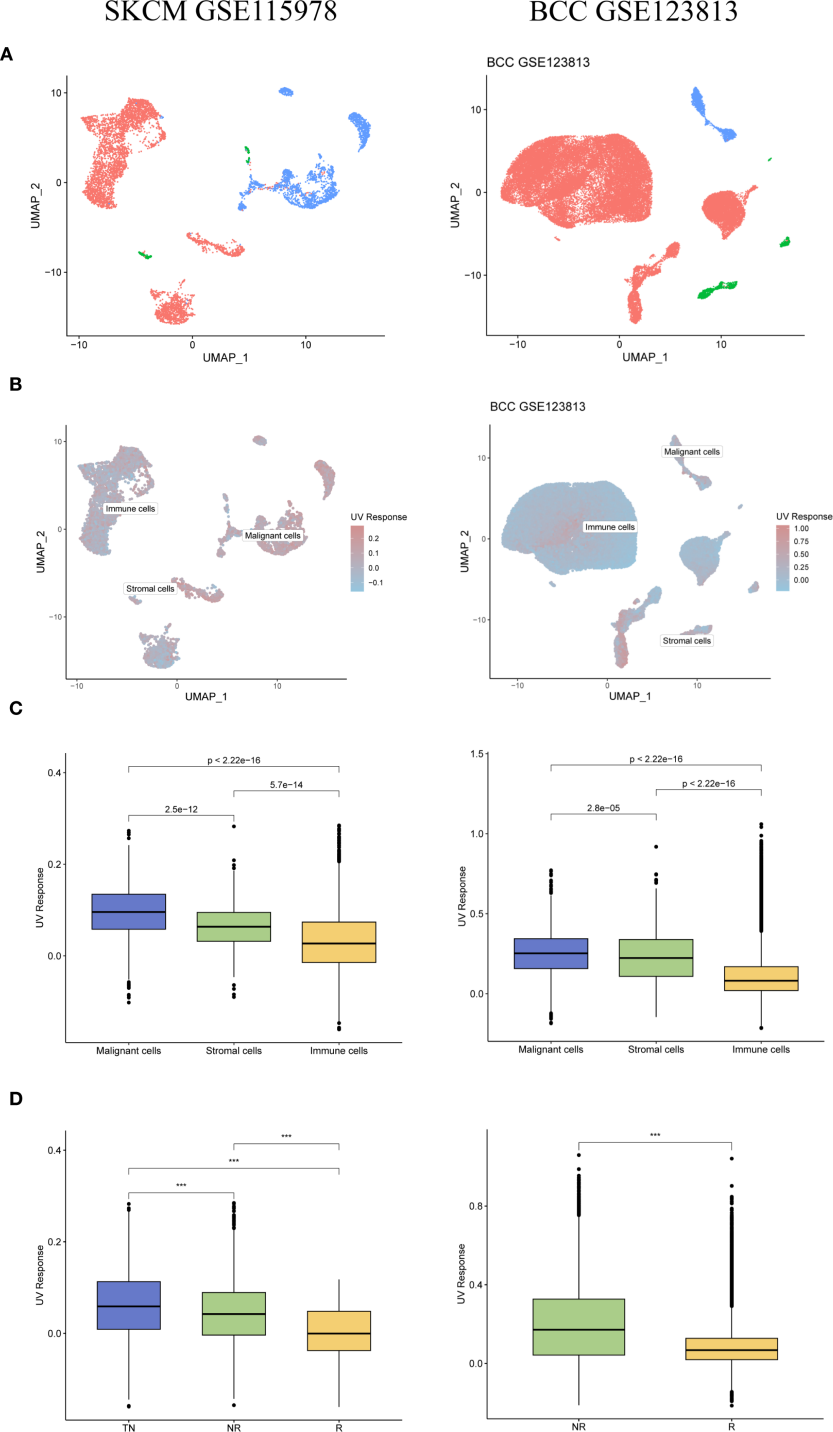
Negative association between UVR-related genes and ICI efficacy in GSE115978 (SKCM) and GSE123813 (BCC). **(A)** Distribution of different cell types within the samples. **(B)** Enrichment scores of UVR-related genes in the samples, with deep red indicating higher scores and deep blue indicating lower scores. **(C)** Differences in UVR scores among various cell types. **(D)** Relationship between immunotherapy efficacy and UVR scores. SKCM, skin cutaneous melanoma; TN, treatment-naive; NR, non-responders; R, responders; ICI, immune checkpoint inhibitor; BCC, basal cell carcinoma. ***indicates a p value < 0.001, denoting highly statistically significant results.

The results from both datasets indicated a significant enrichment of UVR-related genes in malignant tumor cells (**Figures 2A–C**). In the SKCM dataset (GSE115978), the UVR scores of the immunotherapy-effective group (R) were significantly lower than those of the immunotherapy-ineffective group (NR) (p < 0.001) and treatment-naïve group (TN) (p < 0.001). Furthermore, the UVR scores of the NR group were significantly lower than those of the TN group (p < 0.001; **Figure 2D**). Similarly, in the BCC dataset (GSE123813), the UVR scores of the R group were significantly lower than those of the NR group (p < 0.001; **Figure 2D**). These findings suggest a negative correlation between UVR-related genes and ICI efficacy.

### 3.3 Screening and enrichment analysis of UVR-related genes

**Figure 3:**
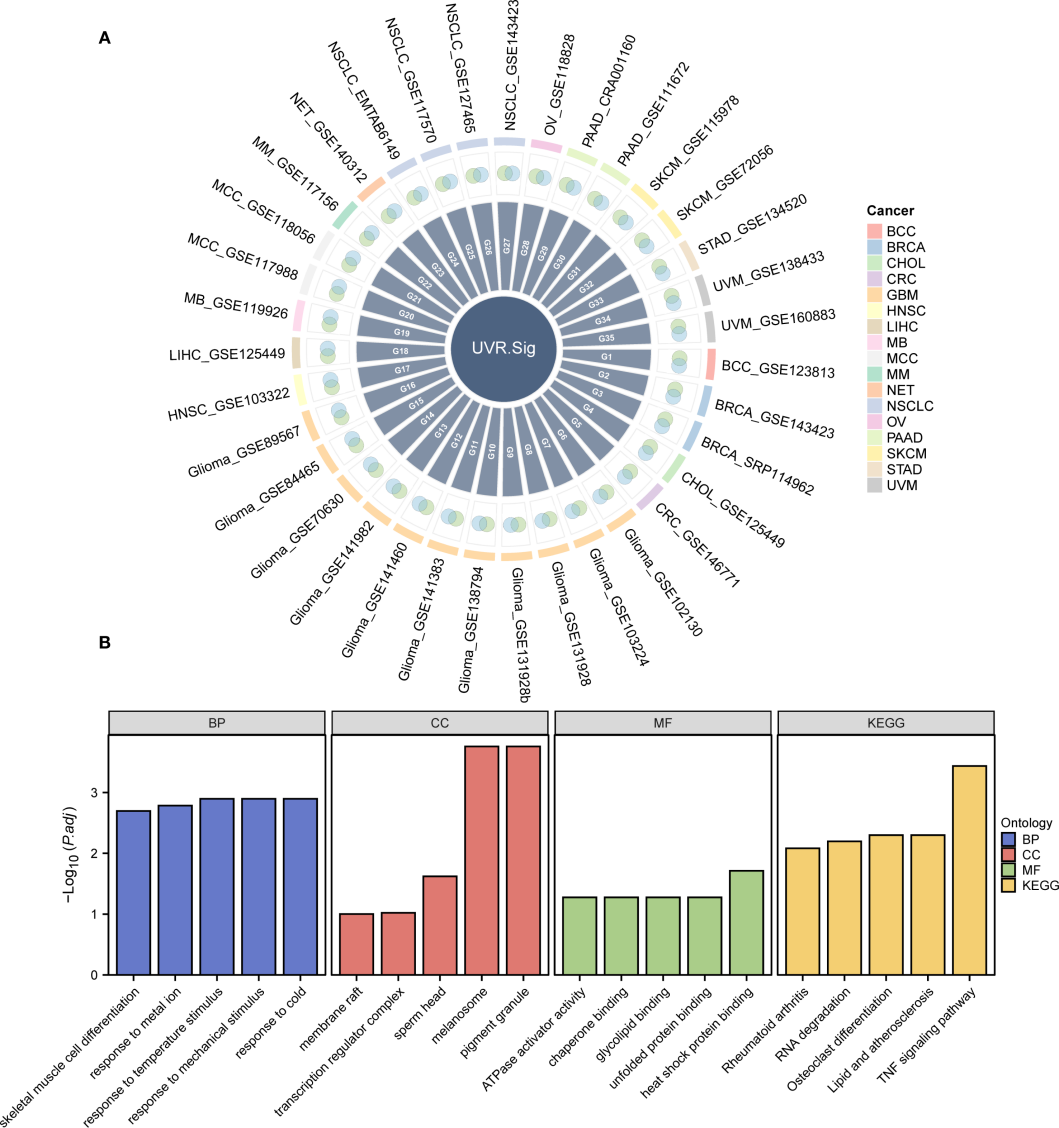
Development and description of UVR.Sig. **(A)** Venn diagram illustrating the intersection of genes positively correlated with UVR enrichment scores across various scRNA-seq datasets and DEGs upregulated in malignant tumor cells. Different colors represent distinct cancer types. **(B)** Bar chart depicting the results of GO and KEGG enrichment analyses for UVR.Sig, categorized into BP, CC, MF, and KEGG. The x-axis represents the GO terms. The selection criteria for GO and KEGG enrichment analyses were adjusted p-value (adj.p) < 0.05 and FDR value (q-value) < 0.25, with p-value adjustments performed using the Benjamini–Hochberg (BH) method. BP, biological process; CC, cellular component; DEG, differentially expressed gene; GO, Gene Ontology; KEGG, Kyoto Encyclopedia of Genes and Genomes; MF, molecular function; scRNA-seq, single-cell RNA sequencing; UVR, ultraviolet response; UVR.Sig, ultraviolet response signature.

Through the analysis of the aforementioned two datasets, a significant association was identified between UVR-related genes and ICI resistance. Consequently, we hypothesized that the expression levels of UVR-related genes in patients could potentially predict the effectiveness of immunotherapy. We obtained 38 up-regulated tumor-specific DEGs in 34 scRNA-Seq datasets that were positively associated with UVR: *ATF3, BTG3, FOS, FOSB, JUNB, NFKBIA, NR4A1, RHOB, SOD2, EPCAM, GGH, IGFBP2, CXCL2, BTG1, DNAJA1, DNAJB1, ALDOA, AP2S1, CDKN1C, ENO2, FEN1, HSPA2, OLFM1, PPIF, BTG2, ICAM1, SQSTM1, AMD1, ATP6V1F, BSG, CNP, CREG1, CYB5R1, MMP14, PPT1, RPN1, SELENOW, STIP1* (**Figure 3A**).

To analyze the BP, MF, and CC, and pathways associated with UVR.Sig, GO and KEGG enrichment analyses were performed, with detailed results provided in **Supplementary Table S2**. The analyses revealed that UVR.Sig was primarily enriched in the following biological processes: response to temperature stimuli, mechanical stimuli, cold, and metal ions; and skeletal muscle cell differentiation. In terms of CC, UVR.Sig was enriched in melanosomes, pigment granules, sperm heads, transcription regulator complexes, and membrane rafts. MF analysis indicated that UVR.Sig was involved in heat shock protein binding, ATPase activator activity, chaperone binding, glycolipid binding, and unfolded protein binding. Furthermore, UVR.Sig was significantly enriched in pathways related to TNF signaling, osteoclast differentiation, lipid and atherosclerosis, RNA degradation, and rheumatoid arthritis signaling pathways. The results of the GO and KEGG enrichment analyses were visualized using bar charts (**Figure 3B**).

### 3.4 Immune correlation analysis of UVR.Sig

**Figure 4:**
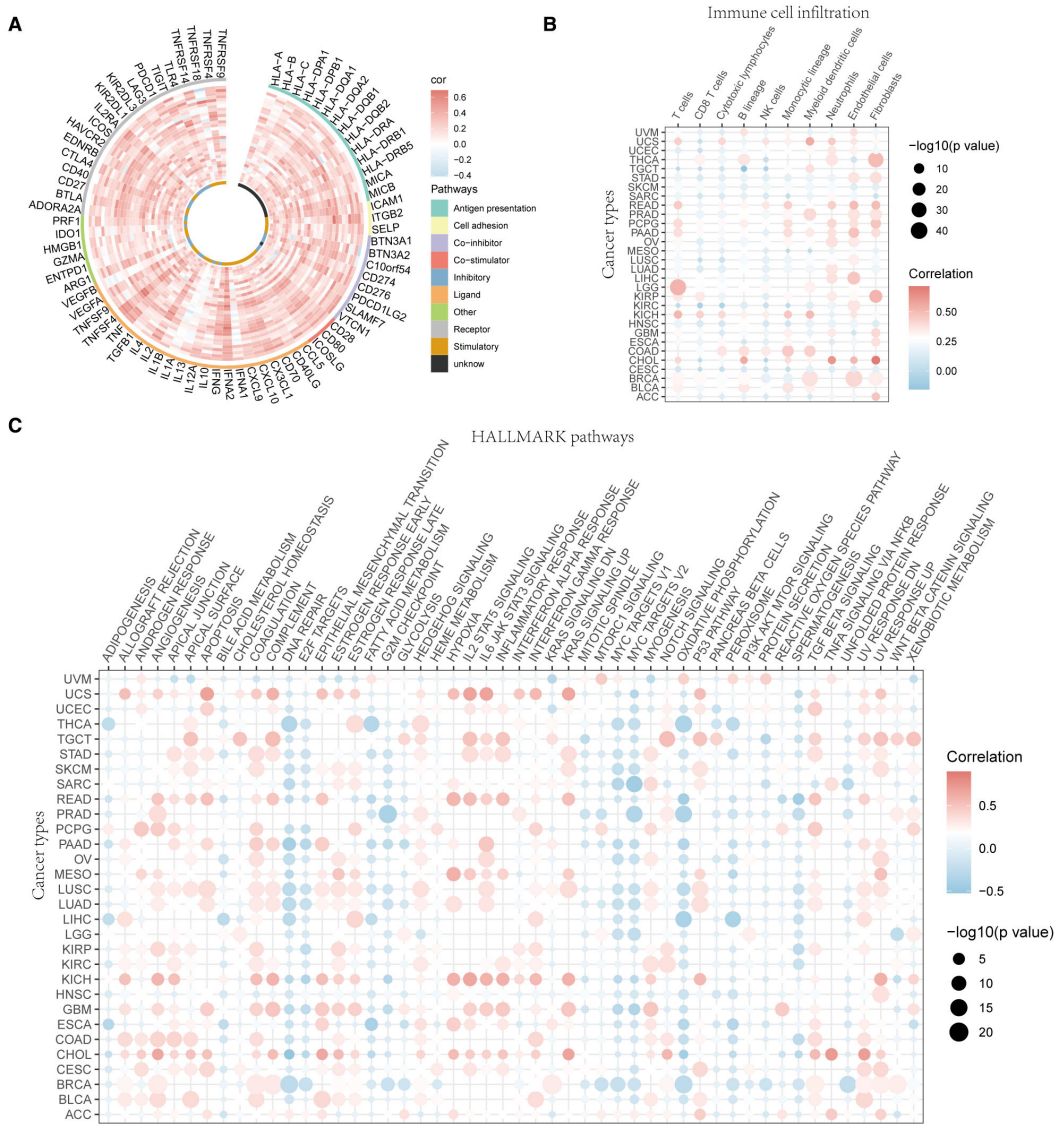
Immune resistance analysis of UVR.Sig. **(A)** Correlation circle diagram between UVR.Sig and immune-related genes. **(B)** Correlation point diagram of UVR.Sig with immune cell infiltration across different cancer types. **(C)** Correlation dot plot of UVR.Sig with HALLMARK pathways across different cancer types. Red indicates positive correlation and blue indicates negative correlation. UVR.Sig, ultraviolet response signature.

The correlation between UVR.Sig and the 75 immune-related genes was analyzed. The results indicated (**Figure 4A**) that UVR.Sig was significantly and positively correlated with most immune genes, suggesting its potential key role in the regulation of immune responses. Subsequently, the infiltration of immune cells across various cancers was assessed, which revealed that the degree of immune cell infiltration associated with UVR.Sig varies among different cancer types (**Figure 4B**). Specifically, in SKCM, HNSC, and mesothelioma (MESO), the UVR.Sig showed a predominantly negative correlation with immune cell infiltration, whereas a positive correlation was observed for CHOL, lower grade glioma (LGG), and kidney chromophobe (KICH). Finally, we examined the relationship between the UVR.Sig and HALLMARK pathways to explore whether immunosuppressive biological functions were upregulated in tumors with high UVR. Sig expression. The results indicated significant enrichment of pathways, such as angiogenesis, epithelial-mesenchymal transition (EMT), Hedgehog signaling, IL-2-STAT signaling, IL-6-JAK-STAT signaling, and inflammatory response in tumors with high UVR.Sig expression (**Figure 4C**). These findings suggest that UVR.Sig regulates immunosuppressive functions within the TME, thereby promoting tumor aggressiveness and resistance.

### 3.5 Construction of immune efficacy prediction model

**Figure 5:**
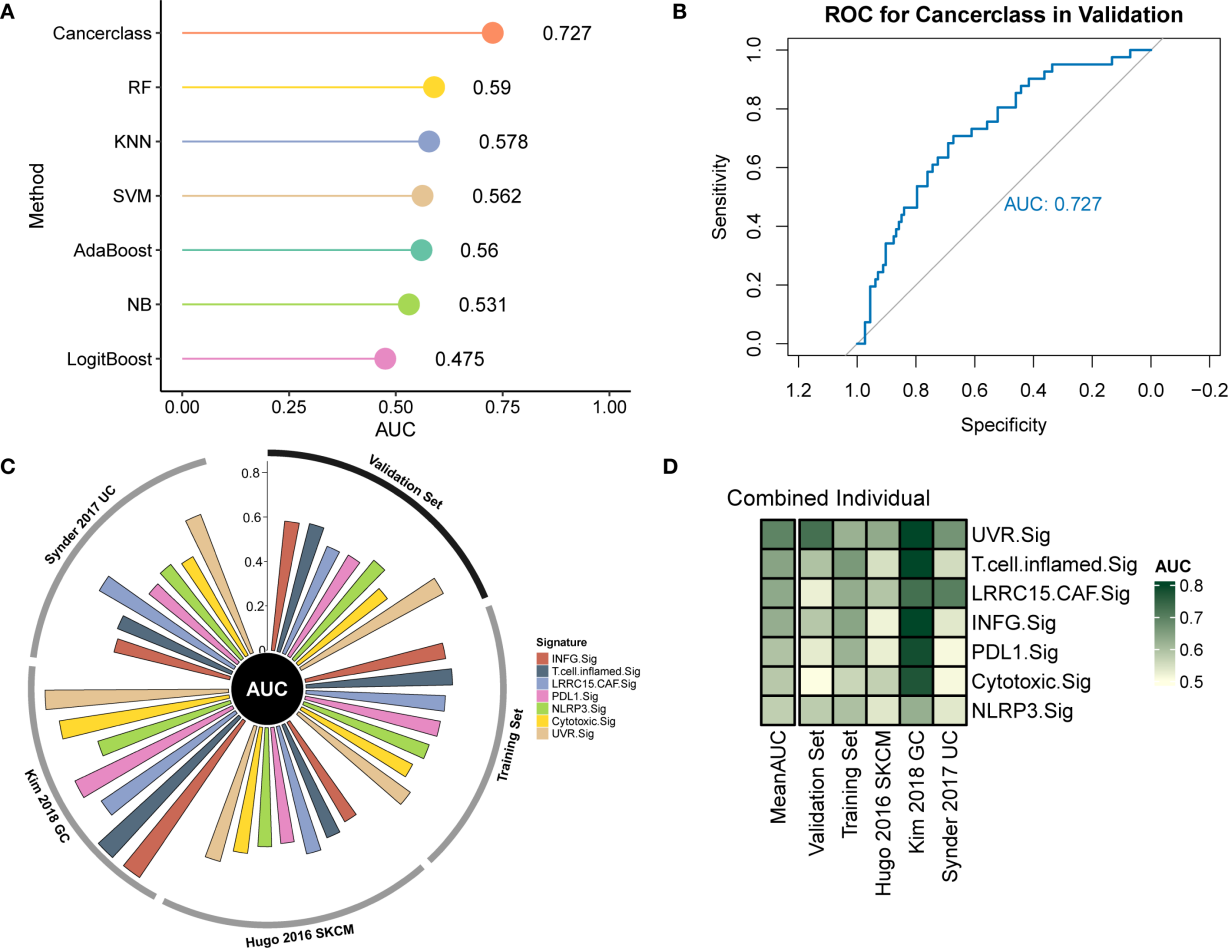
Prediction of immunotherapy outcomes and AUC of UVR.Sig. **(A)** Performance of predictive models for immunotherapy efficacy constructed using seven ML algorithms. **(B)** ROC curve of the prediction model constructed by the Cancerclass algorithm. **(C)** Circle plot comparing the performance of UVR.Sig and other signatures in the training, validation, and testing sets (Hugo 2016 SKCM, Synder 2017 UC, Kim 2018 GC). **(D)** Heatmap comparing the predictive value of UVR.Sig and other signatures. Mean AUC represents the average AUC value. AUC, area under the curve; ML, machine learning; UVR.Sig, ultraviolet response signature.

To investigate the relationship between UVR.Sig and the efficacy of ICIs, we collected 10 RNA-Seq datasets with clearly defined efficacy of immunotherapy, along with their clinical information. All cohorts were divided into a training set (n=618), a validation set (n=154), and five independent testing sets (n=149). First, we constructed immune efficacy prediction models using 7 ML algorithms in the training set, performing 10 rounds of 5-fold cross-validation for parameter optimization. Subsequently, by comparing the AUC values of the 7 models in the validation set, we identified the best-performing model. The results indicated that the model constructed using the Cancerclass algorithm outperformed the others, achieving the highest AUC value of 0.727, and was selected as the UVR.Sig model (**Figures 5A, B**).

Then, we compared the predictive capability of UVR.Sig with 6 other signatures (INFG.Sig, T.cell.infamed.Sig, PDL1.Sig, LRRC15.CAF.Sig, NLRP3.Sig, and Cytotoxic.Sig) across the training, validation, and testing sets. The results demonstrated that UVR.Sig exhibited the best predictive performance in the training set, validation set, and Hugo 2016 SKCM, Snyder 2017 UC, and Kim 2018 GC of the testing sets (**Figure 5C**). In contrast, most previously published signatures only achieved high stability in one or two datasets, while their performance in other external cohorts was considerably unsatisfactory, likely due to poor generalizability. This underscores the potential of UVR.Sig as a pan-cancer predictive model for ICI response. Finally, the heatmap (**Figure 5D**) shows the performance of individual signatures in predicting ICI efficacy across different datasets, further confirming the stability of UVR.Sig.

### 3.6 CRISPR analysis of UVR.Sig

**Figure 6:**
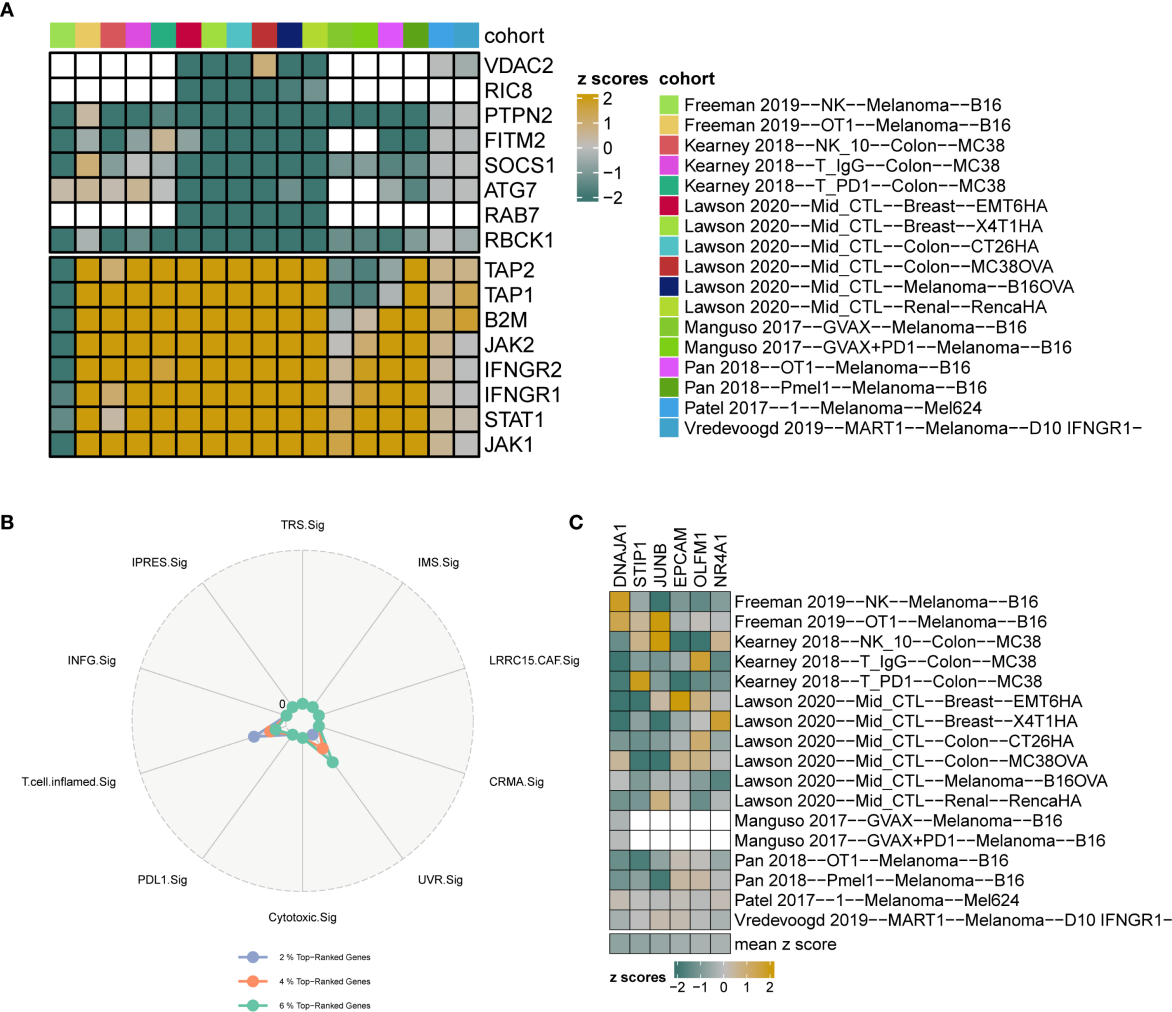
CRISPR analysis of UVR.Sig. **(A)** Gene ranking based on z-scores across 17 CRISPR datasets. Green indicates genes with immune resistance characteristics, where knockout enhances anti-tumor immune function; yellow indicates immune-sensitive genes, where knockout suppresses anti-tumor immunity; white represents missing values for genes in the core data. **(B)** Radar plot comparing the percentage of top-ranking genes from UVR.Sig and nine other signatures. **(C)** Heatmap visualization of the z-scores for six UVR.Sig genes across 17 CRISPR datasets. UVR.Sig, ultraviolet response signature.

Gene-knockout immune response data were systematically collected from seven CRISPR datasets and further categorized into 17 distinct datasets based on the model cells and treatment conditions utilized in each cohort. The 17 CRISPR datasets contained 22,505 genes. Initially, the genes were ranked according to their average Z-scores, with the top-ranking genes identified as potential immune resistance genes, indicating that their knockouts may enhance anti-tumor immunity. Conversely, genes that ranked lower were classified as immune sensitive, which may inhibit the biological functions of anti-tumor immunity upon knockout (**Figure 6A**). Specifically, the top 1%, 2%, and 3% corresponded to 225, 450, and 675 genes, respectively. Subsequently, the percentage of UVR.Sig among the top genes was compared to that of nine other ICI response signatures (INFG.Sig, T.cell.infamed.Sig, PDL1.Sig, LRRC15.CAF.Sig, Cytotoxic.Sig, CRMA.Sig, IPRES.Sig, IMS.Sig, and TRS.Sig). The results indicated that UVR.Sig occupied a higher percentage of the top genes than the other signatures (**Figure 6B**). Notably, among the top 20% of genes, six genes from the UVR.Sig were identified: DNAJA1, STIP1, JUNB, EPCAM, OLFM1, and NR4A1. Validation of the ICI resistance characteristics of these six UVR.Sig genes across 17 CRISPR datasets demonstrated their potential as predictive targets for immunotherapy (**Figure 6C**).

### 3.7 ML-based selection of Hub-UVR.Sig

**Figure 7:**
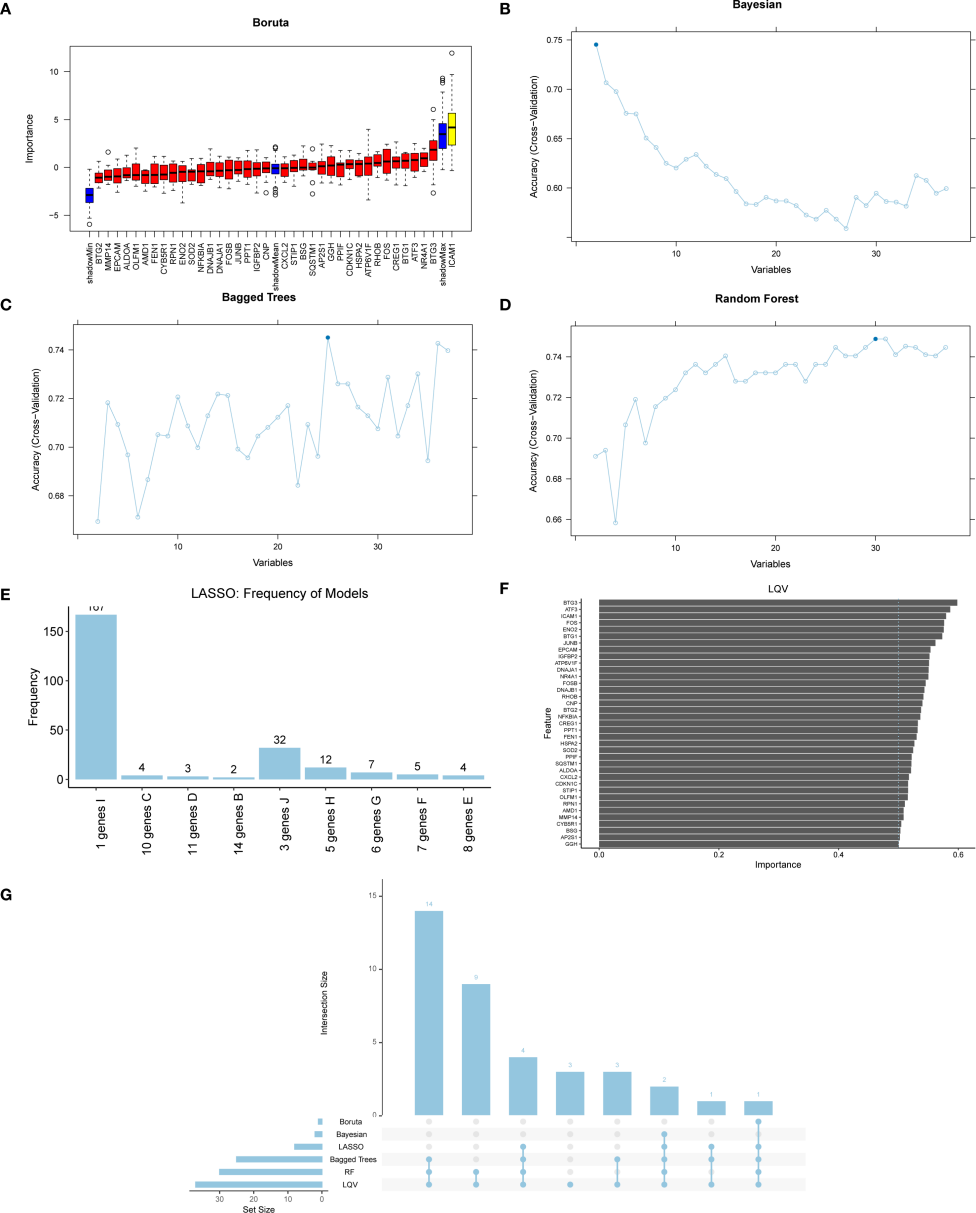
ML and Hub-UVR.Sig analysis. **(A)** Graph of wrapper (Boruta) algorithm prognostic risk model of UVR.Sig. **(B)** Bayesian algorithm prognostic risk model map of UVR.Sig. **(C)** Bagged Trees algorithm prognostic risk model diagram of UVR.Sig. **(D)** Random Forest algorithm prognostic risk model map of UVR.Sig. **(E)** LASSO prognostic risk model map of UVR.Sig. **(F)** Learning vector quantification prognostic risk model plot of UVR.Sig. **(G)** Intersection plot of results from six different ML algorithms. ML, machine learning; UVR.Sig, ultraviolet response signature.

To further refine the UVR.Sig, six ML algorithms were calculated to analyze the relationship between UVR.Sig and ICI efficacy based on the training set: the Wrapper (Boruta) (**Figure 7A**), Bayesian (**Figure 7B**), Bagged Trees (**Figure 7C**), RF (**Figure 7D**), LASSO (**Figure 7E**), and LQV (**Figure 7F**). The intersection of results from different ML algorithms was obtained (**Figure 7G**), selecting genes that appeared in at least four ML algorithms as Hub-UVR.Sig, including *ATF3, ATP6V1F, BTG1, BTG3, ENO2, FOS*, and *ICAM1.*

**Figure 8:**
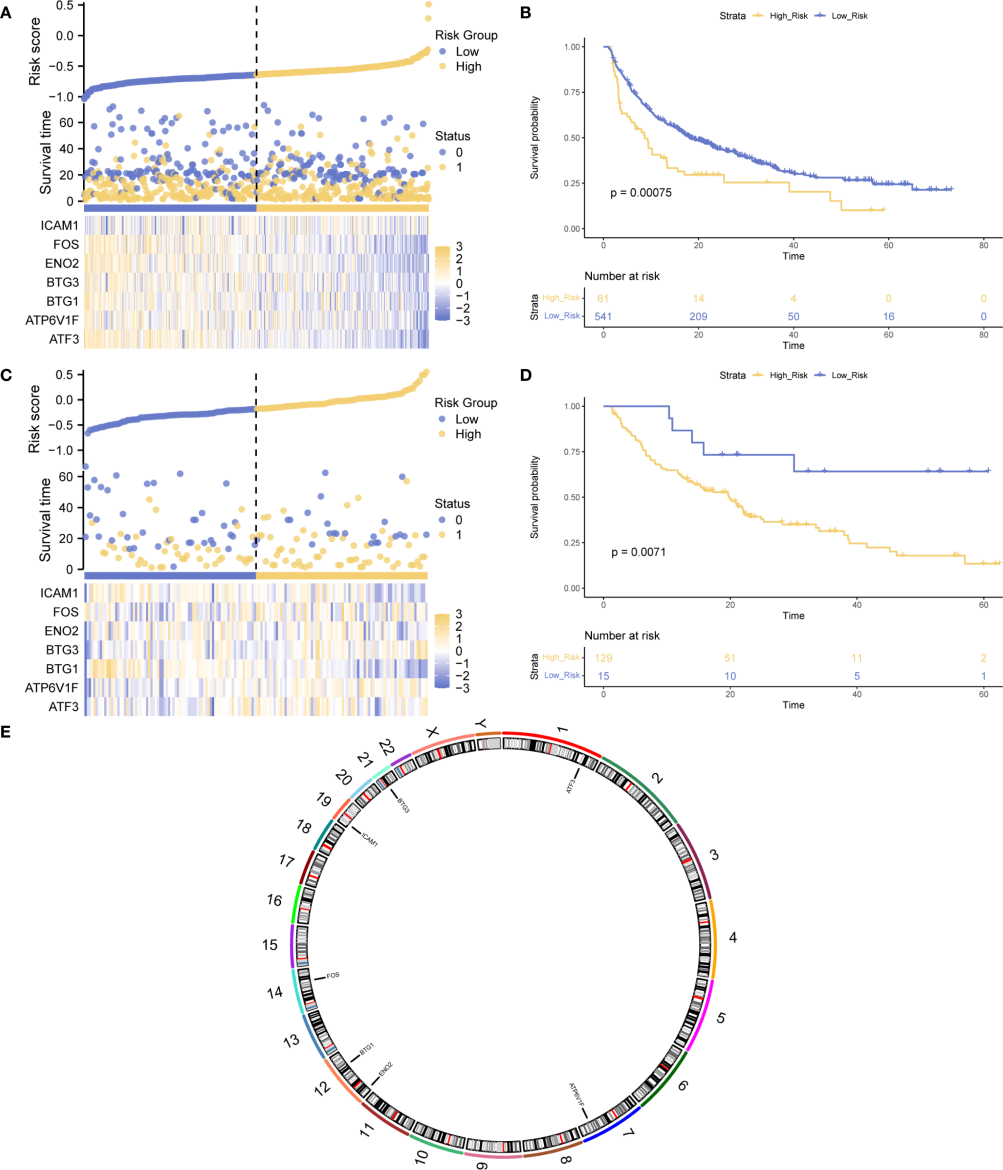
Hub-UVR.Sig and Kaplan–Meier curve analysis. **(A)** Risk factor plot of the Hub-UVR.Sig associated with patient prognosis in the training set. **(B)** Kaplan–Meier curve for high-risk and low-risk patients in the training set. **(C)** Risk factor map of the Hub-UVR.Sig and the prognosis of patients with cancer in the validation set. **(D)** Kaplan–Meier curve for high-risk and low-risk patients in the validation set. **(E)** Chromosomal mapping of the Hub-UVR.Sig. Blue represents the low-risk group, while yellow represents the high-risk group. UVR.Sig, ultraviolet response signature.

To assess the prognostic value of Hub-UVR.Sig, a multivariate Cox regression analysis was performed on the training and validation sets. Risk score was calculated for each sample based on the coefficients of the Cox regression model, and samples were classified into high- and low-risk groups using the “surv_categorize” function, according to the optimal cutoff-value. Risk factor plots were used to visualize the relationships among samples (**Figures 8A, C**), and Kaplan–Meier curve analysis was performed to evaluate the OS (**Figures 8B, D**). The results showed that the low-risk group had significantly better OS in both the training and validation sets compared with the high-risk group (log-rank p < 0.01). Finally, the chromosomal locations of the seven Hub-UVR.Sig genes were analyzed using the RCircos R package, and a chromosome location map was generated (**Figure 8E**). The map revealed that BTG1 and ENO2 were located on chromosome 12.

### 3.8 Panoramic analysis of Hub-UVR.Sig

**Figure 9:**
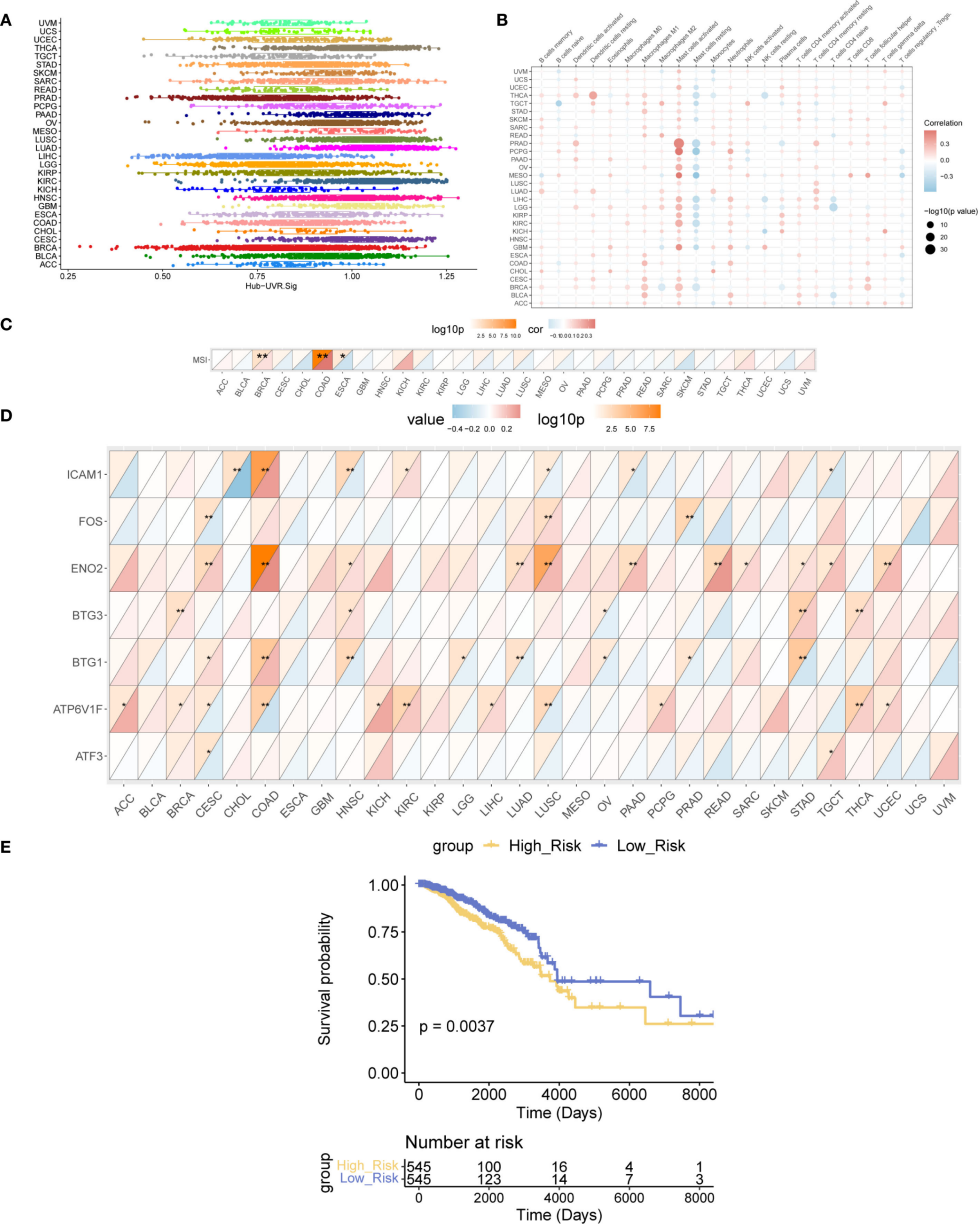
Landscape analysis of Hub-UVR.Sig. **(A)** Scores of the Hub-UVR.Sig across different cancer types. **(B)** Correlation between Hub-UVR.Sig and the abundance of immune cell infiltration in various cancer types. **(C)** Correlation between Hub-UVR.Sig and MSI across 30 cancer types. **(D)** Relationship between the expression levels of Hub-UVR.Sig and MSI in 30 different cancer types. **(E)** Kaplan–Meier curves of the risk scores of the Hub-UVR.Sig and OS in BRCA. The absolute value of the correlation coefficient (r value) is interpreted as follows: values below 0.3 indicate weak or no correlation, values between 0.3 and 0.5 indicate weak correlation, values between 0.5 and 0.8 indicate moderate correlation, and values above 0.8 indicate strong correlation. Red indicates positive correlation, while blue indicates negative correlation. Blue represents the low-risk score group, while yellow represents the high-risk score group. BRCA, breast cancer; MSI, microsatellite instability; OS, overall survival; UVR.Sig, ultraviolet response signature. *p<0.05, **p < 0.01.

The enrichment scores of Hub-UVR.Sig in the TCGA dataset of 30 different cancer types were evaluated using ssGSEA (**Figure 9A**). The correlation between Hub-UVR.Sig and immune cell infiltration was examined in various cancer types. Hub-UVR.Sig showed a significant positive correlation with infiltration of activated dendritic cells, resting dendritic cells, M1 macrophages, M2 macrophages, activated mast cells, and neutrophils. Conversely, it exhibited a significant negative correlation with the infiltration of resting mast cells and naïve CD4+ T cells (**Figure 9B**).

Next, the correlation between Hub-UVR.Sig and MSI was assessed across the 30 cancer types. Hub-UVR.Sig showed the strongest positive correlation with MSI in BRCA and COAD and the strongest negative correlation with ESCA (**Figure 9C**). Furthermore, the relationship between the expression levels of Hub-UVR.Sig and MSI was determined in different cancer types. The results revealed that ICAM1 exhibited the strongest negative correlation with MSI in CHOL, whereas ENO2 showed the strongest positive correlation with MSI in COAD (**Figure 9D**).

Finally, the prognostic ability of Hub-UVR.Sig was analyzed across 30 different cancer types in TCGA cohort. The results of multivariate Cox regression and survival analyses indicated that in BRCA, patients with high-risk scores had significantly worse OS than those with low-risk scores (p < 0.01; **Figure 9E**), suggesting that UVR.Sig may serve as a potential marker for poor prognosis in patients with BRCA.

### 3.9 Construction and correlation analysis of BRCA subtypes

**Figure 10:**
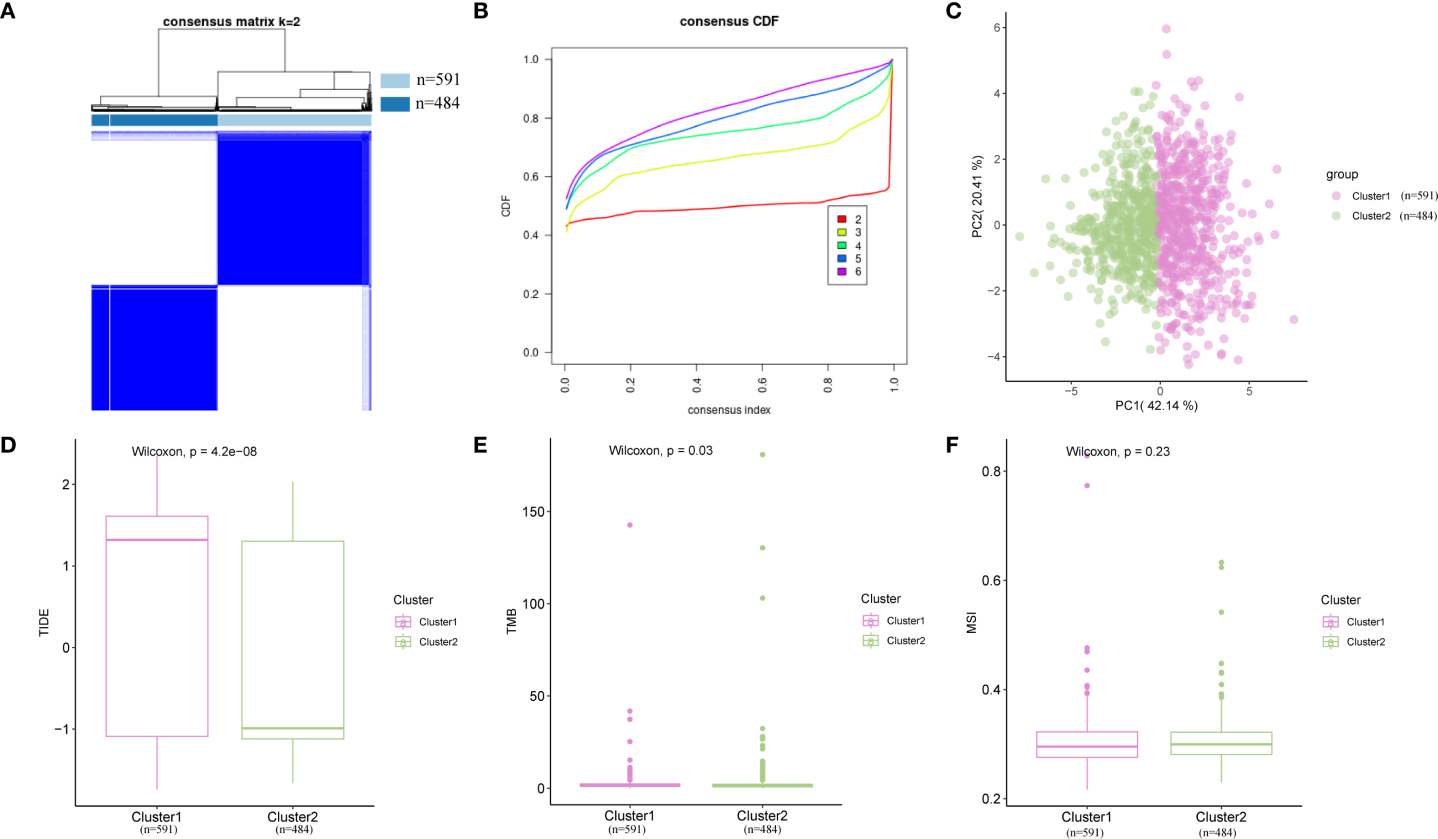
Consensus cluster analysis. **(A)** Consensus cluster analysis results for BRCA samples. **(B)** Consistency cumulative distribution function plot from the consensus clustering analysis. **(C)** PCA plot of two BRCA subtypes. **(D–F)** Comparison of TIDE **(D)**, MSI **(E)**, and TMB **(F)** across different BRCA subtypes. Pink represents subtype A (Cluster 1), while green represents subtype B (Cluster 2). BRCA, breast cancer; MSI, microsatellite instability; PCA, principal component analysis; TIDE, Tumor Immune Dysfunction and Exclusion; TMB, tumor mutational burden.

Given the excellent predictive capability of Hub-UVR.Sig for OS in patients with BRCA, we focused our investigation on its relationship with BRCA. Based on the Hub-UVR.Sig expression levels in TCGA-BRCA samples, a consistency clustering analysis was performed using the R package ConsensusClusterPlus, ultimately identifying two subtypes: BRCA subtypes A (Cluster 1) and B (Cluster 2) (**Figures 10A, B**). Subtype A comprised 591 samples, whereas subtype B comprised 484 samples. The PCA results revealed significant differences between the two subtypes (**Figure 10C**). Further analysis of TIDE (**Figure 10D**), TMB (**Figure 10E**), and MSI scores (**Figure 10F**) in the different BRCA subtypes indicated that there were statistically significant differences in TIDE and TMB scores for the different subtypes, with subtype A showing higher TIDE scores and lower TMB scores compared with subtype B (p < 0.05).

### 3.10 Somatic mutation analysis of BRCA subtypes

**Figure 11:**
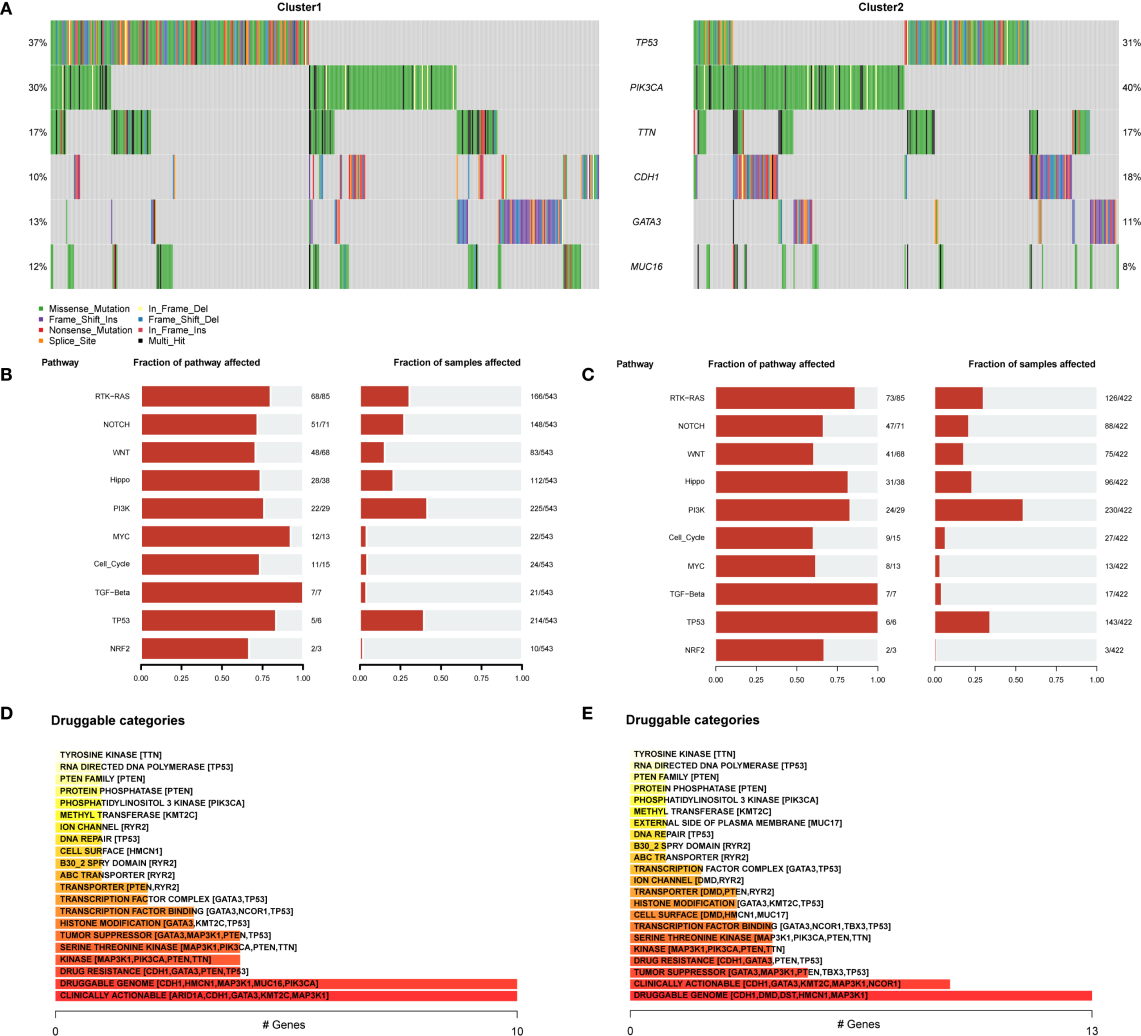
SNP analysis. **(A)** Mutation landscape of BRCA subtype A and subtype **(B)** Each color represents a different type of mutation, with the vertical axis listing the genes with the highest mutation frequencies, including TP53, PIK3CA, TTN, CDH1, GATA3, and MUC16. **(B, C)** Biological function analysis of mutations affecting patients in subtype A **(B)** and B **(C)**. The vertical axis lists various signaling pathways, with the horizontal axis indicating the proportion of samples and pathways affected. **(D, E)** Classification of potentially druggable genes in subtype A **(D)** and B **(E)**. Each classification includes the top five genes in parentheses, and the horizontal axis indicates the number of genes within each druggable gene classification. BRCA, breast cancer.

Subsequently, the mutation frequencies were analyzed in the different BRCA subtypes. The results indicated that the TP53 gene exhibited the highest mutation frequency in subtype A, reaching 37%, while in subtype B, the PIK3CA gene had the highest mutation frequency at 40% (**Figure 11A**). Furthermore, the biological and functional changes induced by mutations were assessed in different subtypes. In subtype A, mutations primarily affected the functions of the TGF-β and PI3K signaling pathways (**Figure 11B**). In contrast, in subtype B, the mutations mainly affected the RTK-RAS and PI3K signaling pathways (**Figure 11C**).

Finally, based on mutation data and the Drug–Gene Interaction database (DGIdb), we explored the gene druggability and drug–gene interactions in patients from different subtypes. As shown in **Figures 11D, E**, in subtype A (Cluster 1), the predicted drugs were potentially targeted at CLINICALLY ACTIONABLE genes including ARID1A, CDH1, GATA3, KMT2C, and MAP3K1; In subtype B (Cluster 2), the predicted drugs were mainly associated with the DRUGGABLE GENOME, involving CDH1, DMD, DST, HMCN1, and MAP3K1.

### 3.11 Immune analysis of BRCA subtypes

**Figure 12:**
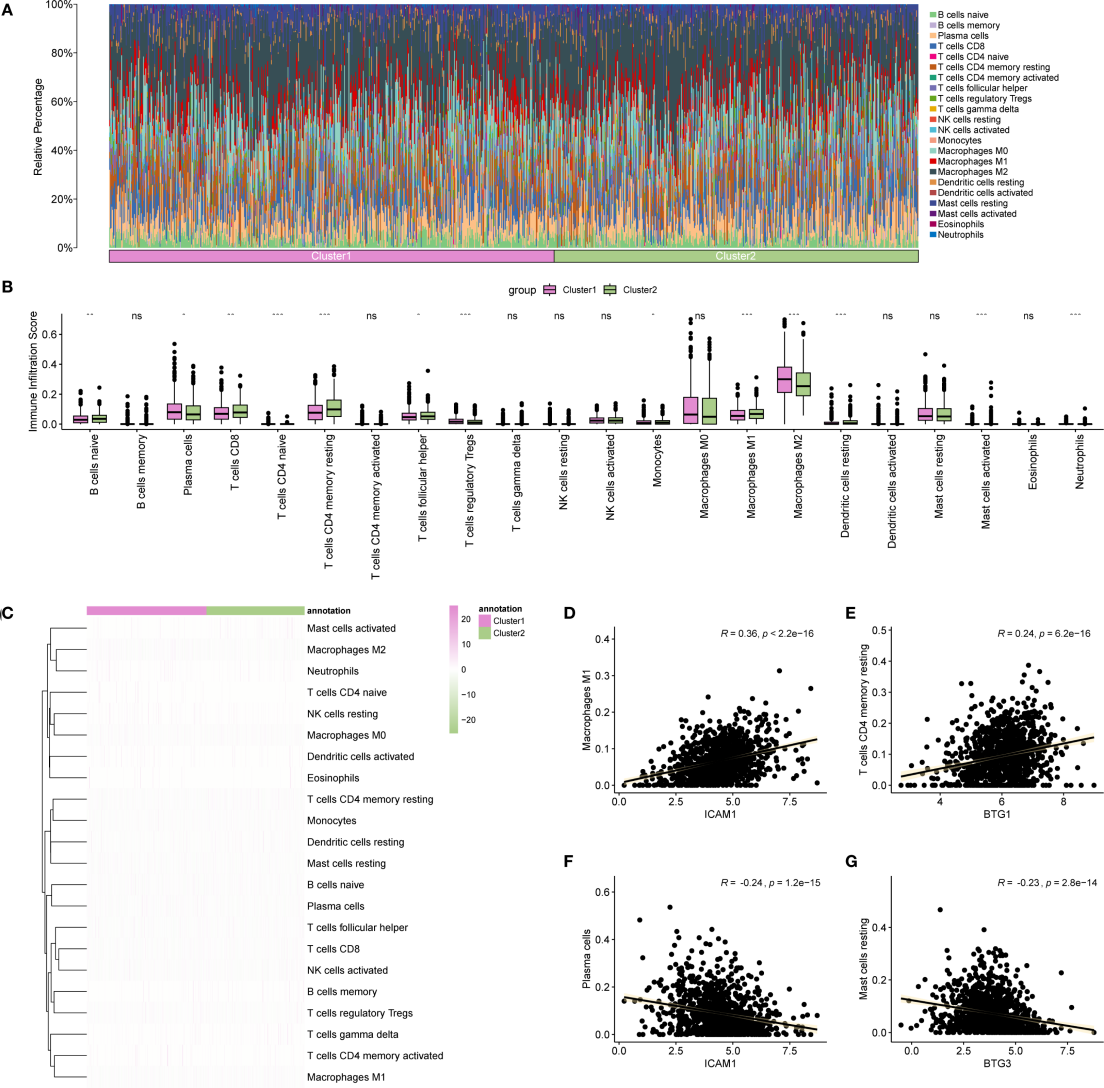
Immune analysis by CIBERSORT algorithm. **(A)** Bar chart showing the proportion of immune cells in different BRCA subtypes. **(B)** Comparison of immune cell infiltration abundance across BRCA subtypes. **(C)** Heatmap illustrating the abundance of various immune cell infiltrations in different BRCA subtypes. **(D–G)** Scatter plots displaying the highest positive and negative correlations between Hub-UVR.Sig and immune cell infiltrations. BRCA, breast cancer; UVR.Sig, ultraviolet response signature. *p<0.05, **p < 0.01, ***p < 0.001, ns: not significant.

The differences in immune cell infiltration were examined across BRCA subtypes. Using the CIBERSORT algorithm, the correlation between 22 immune cell types was assessed within various subtypes and a bar chart illustrating the proportion of immune cells in each BRCA subtype was generated (**Figure 12A**). The results indicated that the enrichment scores for all 22 immune cell types were greater than zero. A grouped comparison chart (**Figure 12B**) was used to highlight the differences in immune cell infiltration abundance among BRCA subtypes. Thirteen immune cell types displayed statistically significant differences in expression levels across subtypes (p < 0.05), including naïve B cells, plasma cells, CD8+ T cells, naïve CD4+ T cells, resting memory CD4+ T cells, follicular helper T cells, Tregs, monocytes, M1 macrophages, M2 macrophages, resting dendritic cells, activated mast cells, and neutrophils. **Figure 12C** presents a heatmap of the infiltration abundance of various immune cells across different BRCA subtypes.

Finally, the correlation between Hub-UVR.Sig and various immune cell infiltrations in BRCA samples were analyzed, focusing on the two pairs with the highest positive and negative correlations (**Figures 12D–G**). The results indicated a positive correlation between ICAM1 and M1 macrophage infiltration scores (r = 0.36; **Figure 12D**). Additionally, BTG1 was positively correlated with resting memory CD4+ T cell infiltration scores (r = 0.24) (**Figure 12E**). Conversely, ICAM1 exhibited a negative correlation with plasma cell infiltration scores (r = -0.24; **Figure 12F**), while BTG3 demonstrated a negative correlation with resting mast cell infiltration scores (r = -0.23; **Figure 12G**).

### 3.12 Expression and function of Hub-UVR.Sig in BRCA

**Figure 13:**
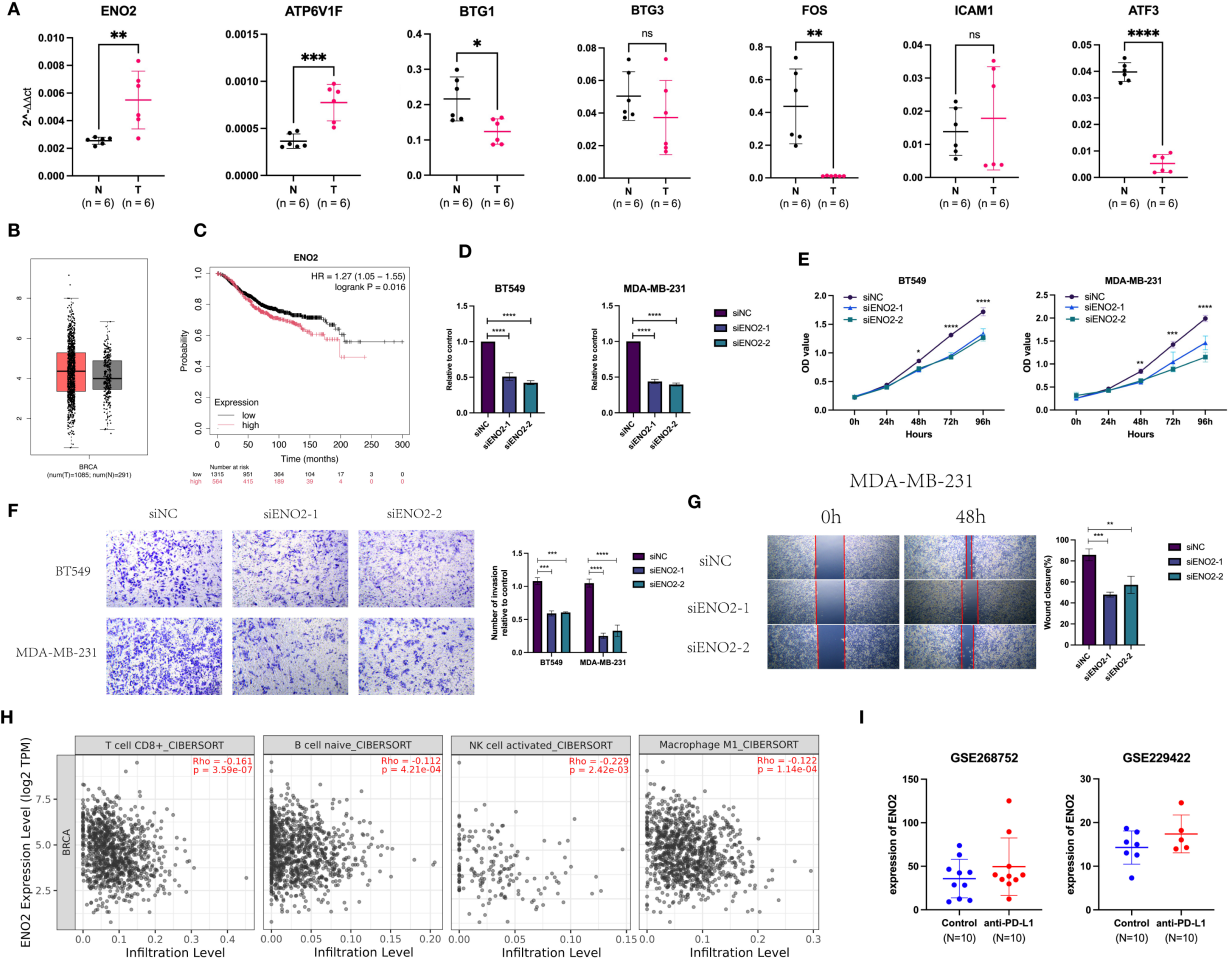
Expression and function of Hub-UVR.Sig in BRCA. **(A)** Hub-UVR.Sig mRNA expression levels in cancer tissues and corresponding paracancerous tissues of six BRCA patients. **(B)** Expression of ENO2 in BRCA tissues and normal tissues. **(C)** ENO2 was associated with worse OS in BRCA patient. **(D)** Knockdown of ENO2 in TNBC cells. **(E)** Knockdown of ENO2 reduced proliferation of TNBC cells. **(F, G)** Knockdown of ENO2 decreased migration **(F)** and invasion **(G)** of TNBC cells. **(H)** Relationship between ENO2 expression and immune infiltrating cells in BRCA. **(I)** Expression of ENO2 increased in mice after treatment with ICIs. BRCA, breast cancer; TNBC, triple-negative breast cancer; UVR.Sig, ultraviolet response signature; *p<0.05, **p < 0.01 ***p < 0.001 and ****p<0.0001. Error bars indicate SD. All results were representative of or combined from at least three independent experiments. ns: not significant.

We examined the mRNA expression levels of Hub-UVR.Sig genes in tumor tissues and paired adjacent normal tissues from six breast cancer patients. The results demonstrated that ENO2 and ATP6V1F were significantly upregulated in tumor tissues (**Figure 13A**). Further analysis of TCGA database revealed that ENO2 expression was markedly higher in breast cancer tissues compared to normal breast tissues (**Figure 13B**). Kaplan-Meier Plotter database (69) indicated that high ENO2 expression was significantly associated with worse OS in breast cancer patients (HR = 1.27, 95% CI = 1.05-1.55, **Figure 13C**).

To investigate the biological functions of ENO2, we conducted experiments using TNBC cell lines MDA-MB-231 and BT549. RT-qPCR confirmed that ENO2-specific siRNA effectively knocked down its expression (**Figure 13D**). CCK8 assays demonstrated that silencing ENO2 reduced the proliferative capacity of tumor cells (**Figure 13E**). Furthermore, ENO2 knockdown inhibited both the migration and invasion abilities of the cells (**Figures 13F, G**). Immune infiltration analysis based on the Timer database (70)showed that ENO2 expression levels were negatively correlated with the infiltration of CD8+ T cells, naive B cells, activated NK cells, and M1 macrophages (**Figure 13H**). Analysis of two GEO datasets (GSE268752 and GSE229422) demonstrated that ENO2 expression was significantly elevated in ICI-treated mice with BRCA compared to control groups (**Figure 13I**).

## 4 Discussion

In this study, we employed the GSVA method to evaluate UVR enrichment scores in malignant cells and found a negative correlation between UVR scores and ICI responses in two scRNA-seq cohorts (SKCM and BCC). Based on these findings, we hypothesized that this negative correlation may be a common phenomenon across various cancer types. To test this hypothesis, we conducted a large-scale comprehensive analysis, identifying a set of genes, termed UVR.Sig, that were highly expressed in malignant cells and significantly positively correlated with UVR scores in 34 scRNA-seq datasets. Rigorous validation of UVR.Sig demonstrated that it outperformed multiple previously used models in predicting ICI responses across bulk RNA-seq independent immunotherapy cohorts. Moreover, the Hub-UVR.Sig, further refined through multiple machine learning methods, showed strong prognostic value for ICI-treated patients, especially in BRCA patients. Based on Hub-UVR.Sig, two distinct BRCA subtypes were identified, each exhibiting unique molecular mutation profiles and immune characteristics, providing a critical foundation for the development of personalized treatment strategies. Finally, by analyzing clinical tissue samples from BRCA patients, we found that ENO2 and ATP6V1F, two genes from the Hub-UVR.Sig, were highly expressed in tumor tissues. High ENO2 expression was associated with worse OS in BRCA patients and negatively correlated with the infiltration of cytotoxic immune cells. Moreover, knockdown of ENO2 significantly suppressed the proliferation, migration, and invasion of TNBC cells.

UV light has been shown to alter the expression of cytokines, chemokines, and cell surface receptors in cells (71–73), thereby modulating various interactions between tumors and the immune system. These interactions may result in either immune activation or immune suppression. For example, UV light can stimulate BRCA cells to secrete pro-inflammatory chemokines, leading to the recruitment of antitumor effector T cells (73). In a CT26 colon cancer mouse model, a single high-dose radiation exposure induced a durable complete remission mediated by CD8^+^ T cell infiltration (74). Conversely, studies have also shown that UV exposure can cause a variety of DNA lesions—including strand breaks, base damage, and cross-linking—which subsequently induce apoptosis in radiation-sensitive tissues such as lymphocytes and result in systemic immunosuppression (75, 76). Furthermore, tumor-associated macrophages exposed to radiation express higher levels of iNOS, arginase-I, and COX-2, thereby promoting tumor growth (77). Therefore, UVR may affect the immune microenvironment in a complex manner, and elucidating the molecular mechanisms by which UV light influences tumor biology is of critical importance.

Our study showed that UVR.Sig, consisting of 38 genes, was mainly enriched in the TNF signaling pathway. In the TME, activated immune cells, such as macrophages and T lymphocytes, fibroblasts, and cancer cells secrete large amounts of TNF-α. This cytokine accumulates within the tumor, triggering and maintaining inflammatory responses that promote tumor growth and progression (78, 79). The TNF-α receptor, TNFR2, is highly expressed in several tumor types, including BRCA, cutaneous T-cell lymphoma, and colorectal cancer (80–82). Overexpression of TNFR2 not only enhances tumor cell resistance to apoptosis, but also inhibits anti-tumor immune responses, thereby facilitating immune evasion (83). Further analysis revealed that UVR.Sig was positively correlated with pathways, such as EMT, hypoxia, IL-6-JAK-STAT3, and inflammatory responses. Hypoxia is a hallmark of the TME (84), that suppresses the activity of T and NK cells, enhances Treg function, and upregulates the expression of immune checkpoint molecules, such as PD-L1 (85, 86). Additionally, STAT3 activation not only upregulates PD-L1 expression (87), but is also associated with the expansion and functional enhancement of various immunosuppressive cells, which secrete factors, such as IL-10, that inhibit effector T cell activity, thereby weakening anti-tumor immune responses (88, 89). Collectively, the association between UVR.Sig and these pathways suggested its potential role in promoting immunosuppression, immune evasion, and the deterioration of the TME.

UVR.Sig was negatively correlated with CD8+ T cells, NK cells, CTLs, and B cells in almost all tumor types, while showing a positive correlation with neutrophils, endothelial cells, and fibroblasts. As key CTLs, CD8+ T and NK cells play critical roles in the immune system by directly killing infected or cancerous cells, thus protecting the body from pathogens and tumors (90–94). Similarly, B cells in the TME recognize tumor-specific antigens and produce antibodies that neutralize tumor cells, thereby preventing their growth and spread of tumor cells, which is closely related to improving the effectiveness of immunotherapy (95, 96). In contrast, a high infiltration of neutrophils, endothelial cells, and fibroblasts in the TME typically indicates immunosuppression (97–102). Tumors with high UVR.Sig exhibited pronounced immunosuppressive characteristics, making them less responsive to ICI therapy. This highlights the potential of UVR.Sig as a valuable predictive marker of immunotherapy response.

Using the optimal Cancerclass algorithm, UVR.Sig was identified as a novel signature that effectively predicted ICI response across various cancer types, including RCC, UC, SKCM, GC, and GBM. To validate its predictive power, UVR.Sig was systematically compared with six widely used pan-cancer signatures. The results demonstrated that UVR.Sig exhibited superior performance (AUC = 0.727) and consistently outperformed other pan-cancer signatures across multiple cancer types and independent cohorts, likely due to its stronger generalizability. In addition, compared with other molecular markers, UVR.Sig may provide a more comprehensive reflection of the overall immune status of the tumor microenvironment. In contrast, PD-L1 primarily reflects surface molecule expression, TMB indicates mutation burden, and MSI reflects genomic stability (103); these markers do not fully capture the complexity of immune cells and signaling pathways within the microenvironment. Therefore, UVR.Sig could represent a complementary tool with potential clinical value in assessing the immunological landscape.

Given the outstanding predictive ability of UVR.Sig for immunotherapy outcomes, the CRISPR dataset was used to identify potential drug targets. This strategy not only revealed novel therapeutic targets but also supported personalized medicine, enabling more precise treatments to enhance efficacy. Based on the correlation between genes and immune response, the genes were ranked and UVR.Sig genes closely associated with immune resistance, including *DNAJA1, STIP1, JUNB, EPCAM, OLFM1*, and *NR4A1*, were identified. DNAJA1, a member of the heat shock protein 40 (Hsp40) family, plays a critical role in regulating B cell function by enhancing the expression and activity of activation-induced cytidine deaminase in mice (104). Additionally, DNAJA1 prevents proteasomal degradation of unfolded mutant p53, thereby promoting tumor metastasis (105). JunB, a member of the activator protein 1 (AP-1) transcription factor family, regulates Treg differentiation, and promotes CD25 expression and IL-2 production (106, 107). JunB plays a pivotal role in immunosuppression and may be a critical factor in predicting adverse reactions to immunotherapy (108). EpCAM, a marker of circulating tumor and cancer stem cells, is expressed in various cancer types (109, 110). It inhibits the activity of CD8+ T cells and upregulates PD-L1 expression, making it a potential immunotherapy target in cancers such as BRCA, COAD, and oral squamous cell carcinoma (111–113). In conclusion, these genes represent potential therapeutic targets across various cancer types, and further investigation of these core UVR.Sig genes will contribute to the development of more effective combination strategies for immunotherapy.

To enhance the prognostic efficacy of UVR.Sig, ML algorithms were employed to identify seven hub genes, termed as Hub-UVR.Sig, including *ATF3, ATP6V1F, BTG1, BTG3, ENO2, FOS*, and *ICAM1*. FOS and JUNB can be directly regulated by p53—p53 binds to the response elements in the promoter region of FOS and promotes its expression. FOS then forms a heterodimer with JUNB, which activates the transcription of downstream immunosuppression-related genes (114). This mechanism may, at least in part, explain the immunosuppressive effect of Hub-UVR.Sig. We observed that Hub-UVR.Sig was positively correlated with activated mast cells and negatively correlated with resting mast cells. Although mast cells are traditionally linked to allergic responses, recent studies have shown that activated mast cells play a critical role in tumor progression and are often associated with poor prognosis (115–117). The mechanisms involved include immune suppression, angiogenesis promotion, and extracellular matrix degradation (118, 119). This suggests that Hub-UVR.Sig may also be involved in the regulation of inflammatory and immune responses by affecting mast cell activation. Risk scores generated based on UVR.Sig are effective in identifying patients with BRCA, and patients with higher risk scores typically exhibit worse OS. This led us to shift our focus to BRCA to further explore the potential value of Hub-UVR.Sig in this context.

Using consensus clustering analysis, two BRCA subtypes that exhibited significant differences in their molecular mutation characteristics and immune infiltration profiles were identified. Subtype A (Cluster 1) had a high mutation frequency in TP53 (37%), whereas Subtype B (Cluster 2) had a high mutation frequency in PIK3CA (40%). TP53 mutations are the most common in BRCA, occurring in 30–35% of all BRCA cases and approximately 80% of triple-negative breast cancer cases (120, 121). Strong evidence has linked TP53 mutations to poor disease-free survival and OS in BRCA (122). PIK3CA mutations are present in 25–46% of BRCA cases and are associated with chemotherapy resistance, poor prognosis, and reduced OS (123). Additionally, Subtype A has a high TIDE score and low TMB score, suggesting a strong immune escape potential and low immunogenicity, potentially leading to a poor response to immunotherapy (65, 124). It should be noted that the consensus clustering in this study was performed at the molecular level and does not fully correspond to pathological BRCA subtypes. Cluster 1 and Cluster 2 were identified through a systematic molecular-level exploration of Hub-UVR.Sig using consensus clustering, whereas pathological classification is mainly based on tumor morphology and conventional biomarkers. Our study therefore represents an initial exploration, and future work will aim to integrate molecular and pathological features to achieve a more comprehensive breast cancer classification.

Finally, we validated the expression of the Hub-UVR.Sig genes in BRCA patients and found that ENO2 and ATP6V1F were highly expressed in tumor tissues. ENO2, a glycolytic enzyme, has been reported to promote stem-like properties, tumorigenesis, and metastatic progression in BRCA cells by activating the glycolytic pathway (125, 126). Consistently, knockdown of ENO2 in TNBC cells resulted in a significant reduction in tumor cell proliferation, migration, and invasion. Moreover, high ENO2 expression was negatively correlated with the infiltration of cytotoxic immune cells and was upregulated following anti-PD-L1 treatment, suggesting that ENO2 may contribute to adaptive ICI resistance.

This study has several limitations. First, the currently available immunotherapy cohorts (GC, SKCM, RCC, UC, and GBM) offer limited tumor type coverage, which may affect the generalizability of our findings. Further validation of UVR.Sig in other cancer types lacking immunotherapy data is warranted, and its broader application across pan-cancer contexts requires support from additional clinical cohorts to ensure robustness and universality. Nevertheless, our immune correlation analysis of UVR.Sig across 30 cancer types in the TCGA partially compensates for this limitation. Second, our study primarily focused on the association between gene expression and response to ICIs, while other key prognostic factors—such as genomic mutations, DNA methylation, histone modifications, and non-coding RNAs—were not considered. The heterogeneity among datasets may also introduce batch effects. Given the robust predictive performance of Hub-UVR.Sig for ICIs response and its prognostic value in BRCA, future studies should prioritize validation using real-world clinical data from BRCA patients, including the prospective collection of clinicopathological information and integration of additional variables that may influence tumor prognosis. We are also aware of the importance of *in vitro* experiments. In the future, we will add functional data verification of Hub-UVR.Sig genes such as ATP6V1F, ICAM1 and BTG family members to further support their biological and clinical relevance and improve the completeness and credibility of this article.

## 5 Conclusion

To our knowledge, this is the first study to reveal a strong association between UVR mechanisms and ICIs resistance in cancer. Through pan-cancer single-cell transcriptomic analysis, we developed a UVR-related gene signature (UVR.Sig) that outperformed existing biomarkers in predicting ICIs response and showed significant prognostic value in breast cancer. While our findings offer a promising tool for refining immunotherapy patient selection, further validation in additional tumor types and incorporation of other prognostic factors are needed to strengthen its clinical applicability.

## Data Availability

The original contributions presented in the study are included in the article/**Supplementary Material**. Further inquiries can be directed to the corresponding author.
