# Supplementary material for: Multi-omics analysis reveals ultraviolet response insights for immunotherapy and prognosis
Source: Front Immunol. 2025 Sep 26;16:1598070. doi: 10.3389/fimmu.2025.1598070 (PMC12510869; doi:10.3389/fimmu.2025.1598070)
Supplement: Supplementary file 3 [file Table2.docx]

**Table S2 Results of GO and KEGG Enrichment Analysis for UVR.Sig**

| ONTOLOGY | ID | Description | GeneRatio | BgRatio | pvalue | p.adjust | qvalue |
| --- | --- | --- | --- | --- | --- | --- | --- |
| BP | GO:0009266 | response to temperature stimulus | 6/38 | 180/18800 | 1.52E-06 | 1.26E-03 | 9.65E-04 |
| BP | GO:0009612 | response to mechanical stimulus | 6/38 | 201/18800 | 2.88E-06 | 1.26E-03 | 9.65E-04 |
| BP | GO:0009409 | response to cold | 4/38 | 51/18800 | 3.31E-06 | 1.26E-03 | 9.65E-04 |
| BP | GO:0010038 | response to metal ion | 7/38 | 351/18800 | 5.71E-06 | 1.64E-03 | 1.25E-03 |
| BP | GO:0035914 | skeletal muscle cell differentiation | 4/38 | 65/18800 | 8.79E-06 | 2.01E-03 | 1.54E-03 |
| CC | GO:0042470 | melanosome | 5/38 | 109/19594 | 2.11E-06 | 1.75E-04 | 1.32E-04 |
| CC | GO:0048770 | pigment granule | 5/38 | 109/19594 | 2.11E-06 | 1.75E-04 | 1.32E-04 |
| CC | GO:0061827 | sperm head | 2/38 | 16/19594 | 4.32E-04 | 2.39E-02 | 1.80E-02 |
| CC | GO:0005667 | transcription regulator complex | 5/38 | 483/19594 | 2.29E-03 | 9.49E-02 | 7.16E-02 |
| CC | GO:0045121 | membrane raft | 4/38 | 326/19594 | 3.56E-03 | 9.95E-02 | 7.50E-02 |
| MF | GO:0031072 | heat shock protein binding | 4/38 | 123/18410 | 1.17E-04 | 1.94E-02 | 1.32E-02 |
| MF | GO:0001671 | ATPase activator activity | 2/38 | 26/18410 | 1.31E-03 | 5.29E-02 | 3.61E-02 |
| MF | GO:0051087 | chaperone binding | 3/38 | 106/18410 | 1.35E-03 | 5.29E-02 | 3.61E-02 |
| MF | GO:0051861 | glycolipid binding | 2/38 | 29/18410 | 1.63E-03 | 5.29E-02 | 3.61E-02 |
| MF | GO:0051082 | unfolded protein binding | 3/38 | 121/18410 | 1.98E-03 | 5.29E-02 | 3.61E-02 |
| KEGG | hsa04668 | TNF signaling pathway | 6/30 | 112/8164 | 2.65E-06 | 3.65E-04 | 3.09E-04 |
| KEGG | hsa04380 | Osteoclast differentiation | 5/30 | 128/8164 | 9.11E-05 | 5.01E-03 | 4.24E-03 |
| KEGG | hsa05417 | Lipid and atherosclerosis | 6/30 | 215/8164 | 1.09E-04 | 5.01E-03 | 4.24E-03 |
| KEGG | hsa03018 | RNA degradation | 4/30 | 79/8164 | 1.84E-04 | 6.34E-03 | 5.37E-03 |
| KEGG | hsa05323 | Rheumatoid arthritis | 4/30 | 93/8164 | 3.45E-04 | 8.26E-03 | 6.99E-03 |

GO，Gene Ontology；KEGG，Kyoto Encyclopedia of Genes and Genomes；BP，Biological Process；CC，Cellular Component；MF，Molecular Function；UVR.Sig，UV Response Signature。
